# The ubiquitin ligase TRIM21 regulates mutant p53 accumulation and gain of function in cancer

**DOI:** 10.1172/JCI164354

**Published:** 2023-03-15

**Authors:** Juan Liu, Cen Zhang, Dandan Xu, Tianliang Zhang, Chun-Yuan Chang, Jianming Wang, Jie Liu, Lanjing Zhang, Bruce G. Haffty, Wei-Xing Zong, Wenwei Hu, Zhaohui Feng

**Affiliations:** 1 Department of Radiation Oncology, Rutgers Cancer Institute of New Jersey, Rutgers, The State University of New Jersey, New Brunswick, New Jersey, USA.; 2 Department of Biological Sciences, Rutgers, The State University of New Jersey, Newark, New Jersey, USA.; 3 Department of Pathology, Princeton Medical Center, Plainsboro, New Jersey, USA.; 4 Department of Chemical Biology, Ernest Mario School of Pharmacy, Rutgers, The State University of New Jersey, Piscataway, New Jersey, USA.

**Keywords:** Cell Biology, Oncology, Cancer, Tumor suppressors, p53

## Abstract

The tumor suppressor *TP53* is the most frequently mutated gene in human cancers. Mutant p53 (mutp53) proteins often accumulate to very high levels in human cancers to promote cancer progression through the gain-of-function (GOF) mechanism. Currently, the mechanism underlying mutp53 accumulation and GOF is incompletely understood. Here, we identified TRIM21 as a critical E3 ubiquitin ligase of mutp53 by screening for specific mutp53-interacting proteins. TRIM21 directly interacted with mutp53 but not WT p53, resulting in ubiquitination and degradation of mutp53 to suppress mutp53 GOF in tumorigenesis. TRIM21 deficiency in cancer cells promoted mutp53 accumulation and GOF in tumorigenesis. Compared with *p53^R172H^* knockin mice, which displayed mutp53 accumulation specifically in tumors but not normal tissues, *TRIM21* deletion in *p53^R172H^* knockin mice resulted in mutp53 accumulation in normal tissues, an earlier tumor onset, and a shortened life span of mice. Furthermore, TRIM21 was frequently downregulated in some human cancers, including colorectal and breast cancers, and low TRIM21 expression was associated with poor prognosis in patients with cancers carrying mutp53. Our results revealed a critical mechanism underlying mutp53 accumulation in cancers and also uncovered an important tumor-suppressive function of TRIM21 and its mechanism in cancers carrying mutp53.

## Graphical abstract

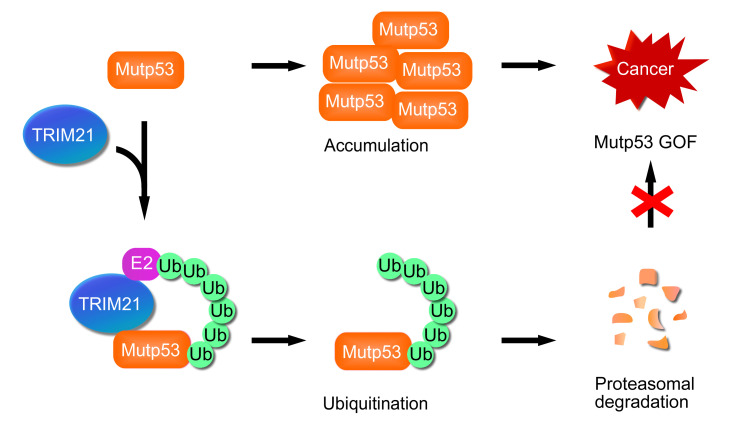

## Introduction

Tumor suppressor p53 plays a central role in tumor suppression, mainly through its transcriptional regulation of a variety of target genes (1–5). The *TP53* (also known as *p53*) gene that encodes the p53 protein is the most frequently mutated gene in human cancers, and its mutations occur in over half of all human cancers (6–9). Intriguingly, the majority of *p53* mutations in cancers are missense mutations, leading to the production of full-length mutant p53 (mutp53) proteins with single amino acid changes. Furthermore, over 85% of these mutations occur in the DNA-binding domain (DBD) of p53, which impairs p53 transcriptional activity. Among them, several hot spot mutations, including mutations at codons 175, 245, 248, and 273, account for approximately 30% of *p53* mutations in cancers (6–10). Tremendous studies have demonstrated that in addition to the loss of the tumor-suppressive function of WT p53 (wtp53), missense mutp53 proteins often display gain-of-function (GOF) activities to promote tumorigenesis independently of wtp53 through different mechanisms (6–18). Interestingly, while wtp53 proteins are kept at low levels under nonstress conditions, mainly through E3 ubiquitin ligase MDM2-mediated ubiquitination and degradation, missense mutp53 proteins often stabilize and accumulate to very high levels in tumors (6–10). Therefore, immunohistochemical (IHC) staining of p53 has been widely used to detect missense *p53* mutations in tumor specimens in clinic, although it is not a perfect marker (10, 19–21). Importantly, mutp53 accumulation in tumors is usually required for mutp53 to exert GOF in tumorigenesis (6, 7, 12, 22–26). Given the high mutational frequency of *p53* in cancer and the GOF activity of mutp53, mutp53 has become an attractive target for cancer therapies (9, 27, 28). Destabilizing mutp53 to inhibit mutp53 GOF activities has been actively tested as a promising therapeutic strategy for cancers carrying mutp53 (9, 13, 22, 27, 28). However, the mechanism underlying mutp53 accumulation in cancer is incompletely understood, and this hinders the development of effective strategies to treat cancers carrying mutp53. Currently, no drugs targeting mutp53 are available for cancer treatment in clinic (9, 27, 28). Therefore, it is crucial to better understand the mechanism underlying mutp53 accumulation and GOF in cancer.

Human tripartite motif (TRIM) family proteins are a large group of proteins (>80 proteins) characterized by the RING, B-Box, and coiled-coil domains at the N-terminus (29–31). Through the RING domain, many TRIM proteins have E3 ubiquitin ligase activity and can ubiquitinate substrate proteins to exert their functions (29–31). TRIM proteins are involved in regulating many biological processes and diseases, including cancer (29–32). Interestingly, several TRIM proteins have been reported to be involved in regulation of wtp53 in a cell/tissue type– and stress-specific manner (33). Recently, TRIM family protein TRIM21 was reported to downregulate wtp53 protein levels indirectly through its regulation of GMP synthase (GMPS) in cells; TRIM21 ubiquitinates GMPS and sequesters it in the cytoplasm, where GMPS cannot promote USP7-mediated deubiquitination of wtp53 in response to DNA damage (34). However, the physiological significance of this regulation in vivo remains unclear, and the effect of TRIM21 on mutp53 is unknown. Emerging evidence has suggested that TRIM21 is involved in cancer. Interestingly, TRIM21 was reported to play a tumor-suppressive role in some cancers (e.g., breast and colorectal cancer) (35–37), whereas it was reported to promote tumorigenesis in some other cancers (e.g., brain and liver cancer) (38, 39). Currently, the precise role and mechanism of TRIM21 in cancer remain elusive.

In this study, we screened for specific mutp53-interacting proteins using coimmunoprecipitation (co-IP), followed by liquid chromatography–tandem mass spectrometry (LC-MS/MS) analysis. We identified mutp53 as a direct substrate for TRIM21; TRIM21 specifically bound to mutp53 but not wtp53, leading to mutp53 ubiquitination and degradation. TRIM21 deficiency promoted mutp53 accumulation and GOF in tumorigenesis in orthotopic and s.c. xenograft tumor models as well as the hot spot R172H mutp53–knockin (mouse R172H mutp53 is equivalent to human R175H) mice. These results reveal a critical mechanism for mutp53 accumulation and GOF in cancer and the tumor-suppressive function of TRIM21 in cancers carrying mutp53.

## Results

### TRIM21 is a mutp53-interacting protein.

To investigate the mechanism underlying mutp53 accumulation in cancer, we screened for specific mutp53-interacting proteins using co-IP with a p53 antibody, followed by LC-MS/MS analysis in human colorectal cancer *p53^−/−^* RKO cells expressing ectopic hot spot R175H mutp53 or wtp53. This approach led to the identification of a list of potential R175H mutp53-interacting proteins, including some known mutp53-interacting proteins, such as several heat shock proteins and BAG2 (10, 13, 22, 40). Interestingly, TRIM21 was identified as a potential protein preferentially binding to R175H mutp53 but not wtp53 (Supplemental Table 1; supplemental material available online with this article; https://doi.org/10.1172/JCI164354DS1).

**Figure 1:**
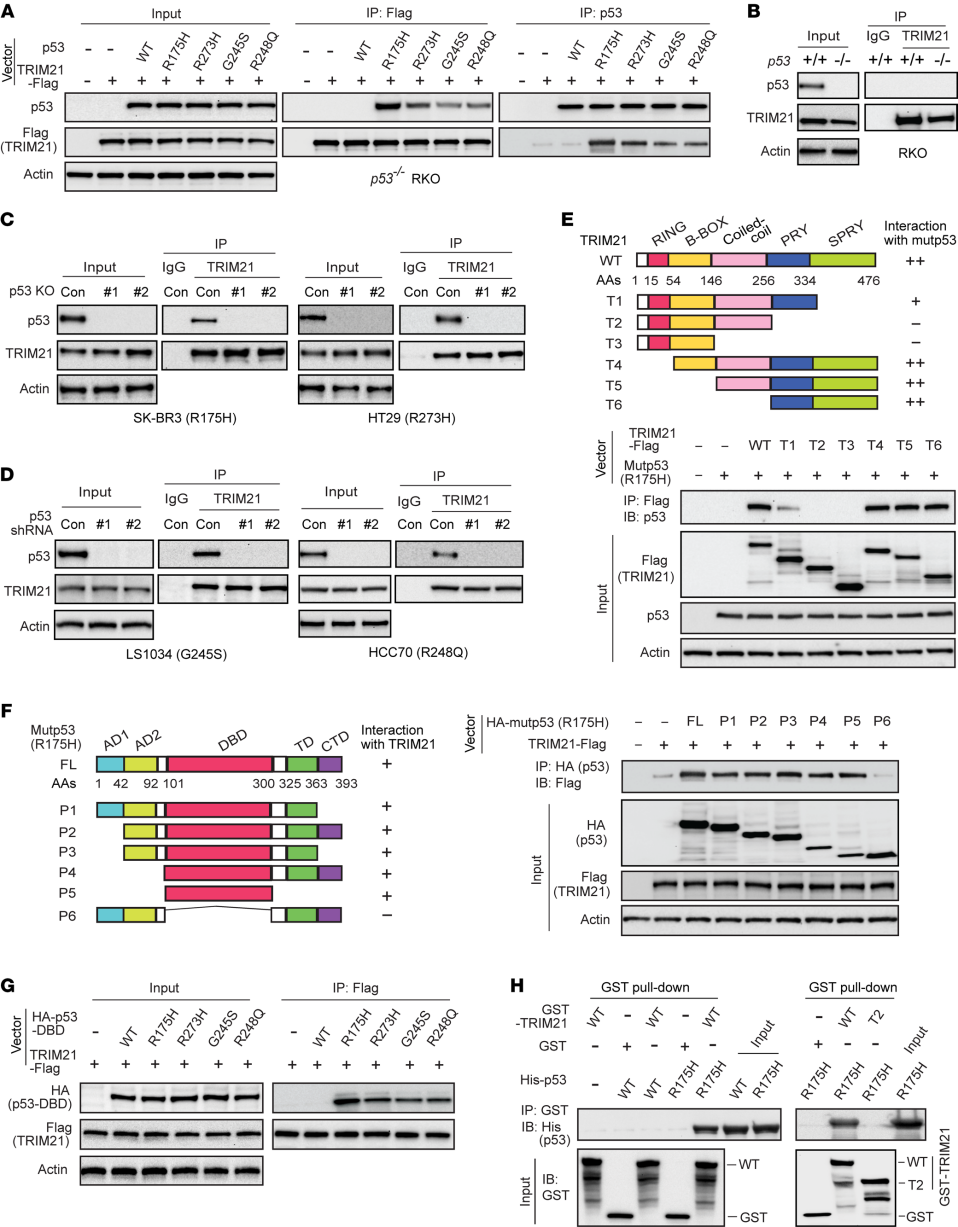
TRIM21 is a specific mutp53-binding protein. (**A**) TRIM21 protein specifically interacted with hot spot mutp53 proteins but not wtp53 in cells. *p53^−/−^* RKO cells expressing ectopic wtp53 or different mutp53 together with TRIM21-Flag were employed for co-IP assays. Control vectors are indicated by dashes. (**B**) Co-IP analysis of the interaction of endogenous TRIM21 with wtp53 in *p53^+/+^* RKO cells. *p53^−/−^* RKO cells were used as negative controls. (**C**) Co-IP analysis of the interaction of endogenous TRIM21 with R175H and R273H mutp53 in SK-BR3 and HT29 cells, respectively. Control (Con) cells and 2 individual clonal cell lines with mutp53 KO by CRISPR/Cas9 were used. (**D**) Co-IP analysis of the interaction of endogenous TRIM21 with G245S and R248Q mutp53 in LS1034 and HCC70 cells, respectively. Cells with mutp53 knockdown by 2 different shRNA vectors were used as negative controls. (**E**) The PRY and SPRY domains of TRIM21 are required for R175H mutp53-TRIM21 interaction. Top: Schematic representation of vectors expressing WT and serial deletion mutants of TRIM21-Flag. Bottom: *p53^−/−^* RKO cells expressing R175H mutp53 and WT or mutant TRIM21-Flag were used for co-IP assays. (**F**) The DBD domain of R175H mutp53 is required for mutp53-TRIM21 interaction. Left: Schematic representation of vectors expressing full-length (FL) and serial deletion mutants of R175H HA-mutp53. AD, transactivation domain; DBD, DNA-binding domain; TD, tetramerization domain; CTD, C-terminal domain. Right: *p53^−/−^* RKO cells expressing TRIM21-Flag and FL or deletion mutants of R175H HA-mutp53 were used for co-IP assays. (**G**) The DBD domain of different hot spot mutp53 interacted with TRIM21-Flag in *p53^−/−^* RKO cells analyzed by co-IP assays. (**H**) The direct interaction of recombinant R175H His-mutp53 with WT but not T2 mutant GST-TRIM21 proteins analyzed by in vitro GST pull-down assays. IP, immunoprecipitation; IB, immunoblotting.

*p53* missense mutations have two major categories: conformational (e.g., R175H and G245S) and DNA-contact mutations (e.g., R248Q and R273H) (10). R175H, G245S, R248Q, and R273H hot spot mutp53 have been shown to display GOF activities and have been widely used for mutp53 GOF studies (6–8, 10). Here, we examined the interaction between TRIM21 and the 4 hot spot GOF mutp53 using co-IP, followed by Western blot assays in *p53^−/−^* RKO cells expressing ectopic TRIM21-Flag and mutp53 (Figure 1A). TRIM21-Flag interacted with these 4 mutp53 and its interaction with R175H mutp53 appeared to be a little stronger (Figure 1A). In contrast, no interaction between TRIM21-Flag and wtp53 was observed, which is consistent with a previous study (34). The interaction between endogenous TRIM21 and mutp53 was confirmed by co-IP assays in different human cancer cell lines expressing different homozygous endogenous GOF mutp53, including breast cancer SK-BR3 (R175H) and HCC70 (R248Q) cells as well as colorectal cancer HT29 (R273H) and LS1034 (G245S) cells. While no clear interaction between the endogenous TRIM21 and wtp53 was observed in *p53^+/+^* RKO cells (Figure 1B), the interaction between the endogenous TRIM21 and different mutp53 was observed in above-mentioned cell lines (Figure 1, C and D). SK-BR3 and HT29 cells with KO of mutp53 using CRISPR/Cas9 technique as well as LS1034 and HCC70 cells with mutp53 knockdown by different shRNA vectors were employed as negative controls for assays to confirm the specificity of this interaction (Figure 1, C and D).

To determine the domain(s) of TRIM21 required for the TRIM21-mutp53 interaction, vectors expressing serial deletion mutants of TRIM21-Flag (Figure 1E) were constructed and cotransfected with R175H mutp53 vectors into *p53^−/−^* RKO cells for co-IP assays. SPRY (SPIa and the ryanodine receptor) and PRY (SPRY-associated) domains are known to be crucial for many TRIM family proteins, including TRIM21, to interact with other proteins (29–31). Our results showed that while deletion of the SPRY domain dramatically reduced the TRIM21-R175H mutp53 interaction, deletion of both SPRY and PRY domains completely abolished the interaction. Furthermore, SPRY and PRY domains were sufficient for TRIM21 to interact with R175H mutp53 (Figure 1E). Our results further showed that the DBD domain of R175H mutp53 was required and sufficient for R175H mutp53 to interact with TRIM21 (Figure 1F). In addition to R175H mutp53, the DBD domain of R273H, G245S and R248Q mutp53 also interacted with TRIM21 in cells (Figure 1G). Results from in vitro glutathione *S*-transferase (GST) pull-down assays using recombinant GST-TRIM21 and His-mutp53 or His-wtp53 proteins showed that TRIM21 directly interacted with mutp53 but not wtp53 in vitro (Figure 1H). Furthermore, deletion of SPRY and PRY domains abolished the ability of GST-TRIM21 to directly interact with His-mutp53 in vitro (Figure 1H). Collectively, these results demonstrate that TRIM21 directly and specifically interacts with mutp53 in cells.

### TRIM21 suppresses mutp53 accumulation in cancer cells.

**Figure 2:**
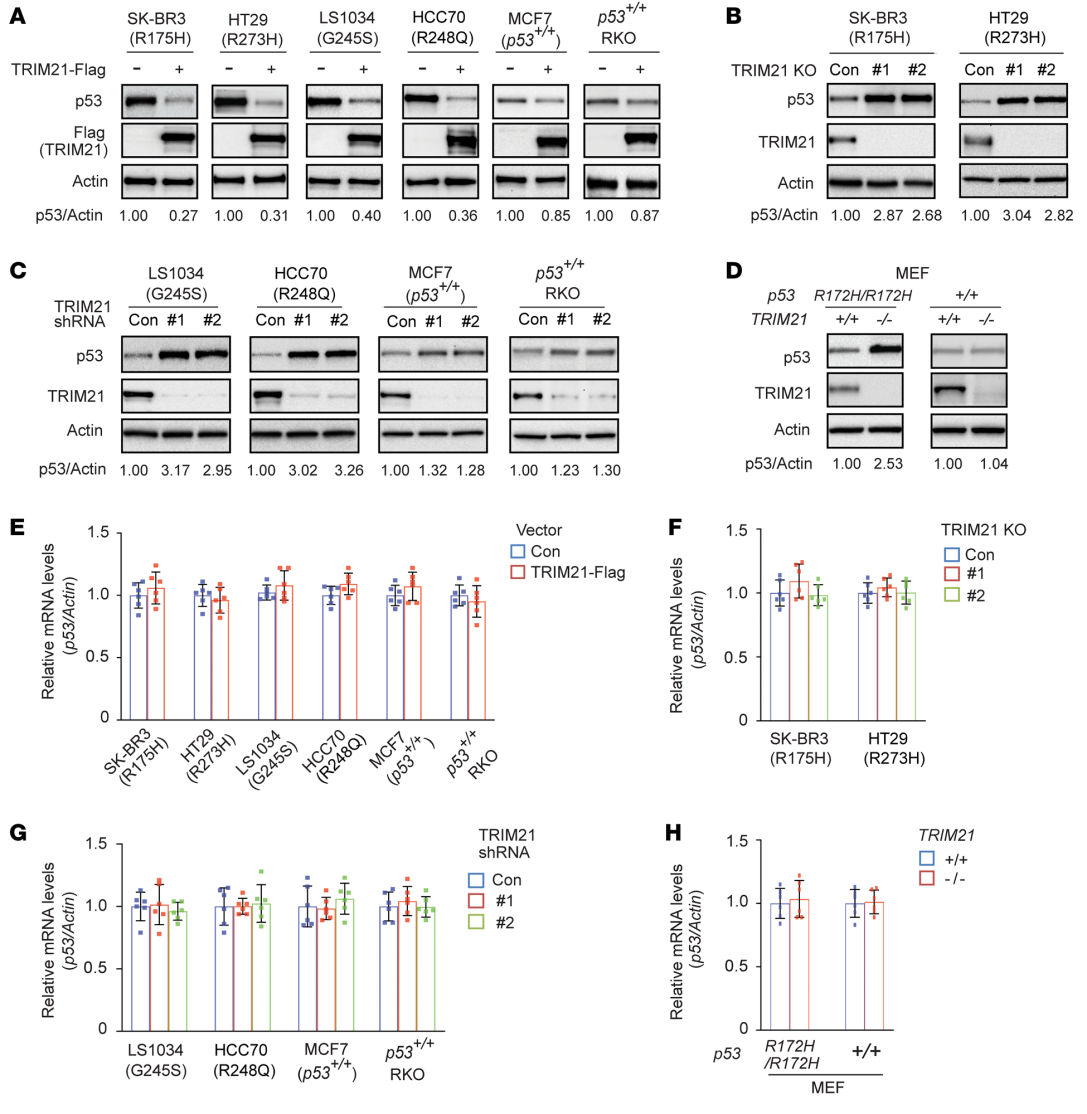
TRIM21 downregulates mutp53 protein levels in cells. (**A**) The effect of ectopic TRIM21 expression on the levels of different endogenous mutp53, including R175H, G245S, R248Q, and R273H, as well as wtp53 proteins in different human cancer cells. Cells were transduced with control (–) or TRIM21-Flag (+) expression vectors for Western blot assays. (**B**) The effect of TRIM21 KO on levels of R175H and R273H mutp53 protein in SK-BR3 and HT29 cells, respectively, analyzed by Western blot assays. (**C**) The effect of knockdown of endogenous TRIM21 by shRNA vectors on the levels of G245S and R248Q mutp53 in LS1034 and HCC70 cells, respectively, and wtp53 proteins in MCF7 and *p53^+/+^* RKO cells. Cells were transduced with control (Con) vectors or 2 different shRNA vectors against TRIM21 for Western blot assays. (**D**) Higher mutp53 protein levels in *TRIM21^−/−^ p53^R172H/R172H^* MEFs compared with *TRIM21^+/+^ p53^R172H/R172H^* MEFs analyzed by Western blot assays. (**E**) Ectopic TRIM21-Flag expression did not affect the mRNA levels of *mutp53* or *wtp53* in different human cancer cells. (**F**) TRIM21 KO did not affect *mutp53* mRNA levels in SK-BR3 and HT29 cells. (**G**) TRIM21 knockdown by shRNA vectors did not affect the mRNA levels of *mutp53* or *wtp53* in different human cancer cells. (**H**) *TRIM21* loss did not affect the mRNA levels of *R172H mutp53* in *p53^R172H/R172H^* MEFs or *wtp53* in *p53^+/+^* MEFs. Mouse R172H mutp53 is equivalent to human R175H mutp53. In **E**–**H**, the mRNA levels of *mutp53* or *wtp53* in cells were measured by TaqMan real-time PCR assays and normalized with *Actin*. The TRIM21 did not show significant effect on *mutp53* or *wtp53* mRNA levels in these cells. Data are shown as the mean ± SD (*n* = 6). Statistical analysis was performed using 2-tailed unpaired Student’s *t* test (**E** and **H**) or 1-way ANOVA followed by Dunnett’s test (**F** and **G**).

Given that TRIM21 is an E3 ubiquitin ligase and interacts with mutp53, we tested whether TRIM21 downregulates mutp53 protein levels in cells. Ectopic expression of TRIM21-Flag greatly downregulated the levels of different endogenous mutp53 proteins in SK-BR3, HT29, LS1034, and HCC70 cells (Figure 2A). Notably, compared with its effect on mutp53, TRIM21-Flag displayed a much less pronounced inhibitory effect on wtp53 protein levels in cells expressing endogenous wtp53, including *p53^+/+^* RKO and human breast cancer MCF7 cells (Figure 2A), which could be through the reported indirect regulation by TRIM21 (34). Furthermore, TRIM21 KO clearly increased the protein levels of R175H and R273H mutp53 in SK-BR3 and HT29 cells, respectively (Figure 2B). Similarly, TRIM21 knockdown by different shRNA vectors clearly increased G245S and R248Q mutp53 protein levels in LS1034 and HCC70 cells, respectively, but displayed a much less pronounced effect on wtp53 protein levels in MCF7 and *p53^+/+^* RKO cells (Figure 2C). R172H mutp53–knockin mice have been widely used for mutp53 GOF studies (41, 42). Employing mouse embryonic fibroblasts (MEFs) derived from *TRIM21^+/+^ p53^R172H/R172H^* and *TRIM21^–/–^ p53^R172H/R172H^* mice, we found that *TRIM21^–/–^ p53^R172H/R172H^* MEFs displayed much higher mutp53 protein levels than *TRIM21^+/+^ p53^R172H/R172H^* MEFs (Figure 2D). In contrast, no obvious difference in wtp53 protein levels was observed between *TRIM21^+/+^ p53^+/+^* and *TRIM21^–/–^ p53^+/+^* MEFs (Figure 2D). Results from TaqMan real-time PCR assays showed that TRIM21 did not clearly affect mRNA levels of *mutp53* or *wtp53* in above-mentioned cell lines (Figure 2, E–H). It has been reported that regulation of gene expression is an important mechanism for mutp53 to exert its GOF (10, 43). Therefore, we investigated whether TRIM21 affects mutp53 function in terms of the regulation of gene expression through its downregulation of mutp53 levels using real-time PCR assays. Knockdown of R175H mutp53 by shRNA significantly reduced the expression of several well-known mutp53-regulated genes, including *CXCL1*, *IGFBP3*, *NFKB2*, and *P2RX5* (10, 43–45), in the SK-BR3 cells (Supplemental Figure 1). Notably, TRIM21 knockdown by shRNA clearly induced the expression of these genes, which was largely abolished by mutp53 knockdown, suggesting that TRIM21 negatively regulates the function of mutp53 in gene regulation (Supplemental Figure 1). These results indicate that TRIM21 preferentially downregulates protein levels of mutp53 but not wtp53, and TRIM21 deficiency leads to mutp53 accumulation in cancer cells.

### TRIM21 promotes ubiquitination and degradation of mutp53.

**Figure 3:**
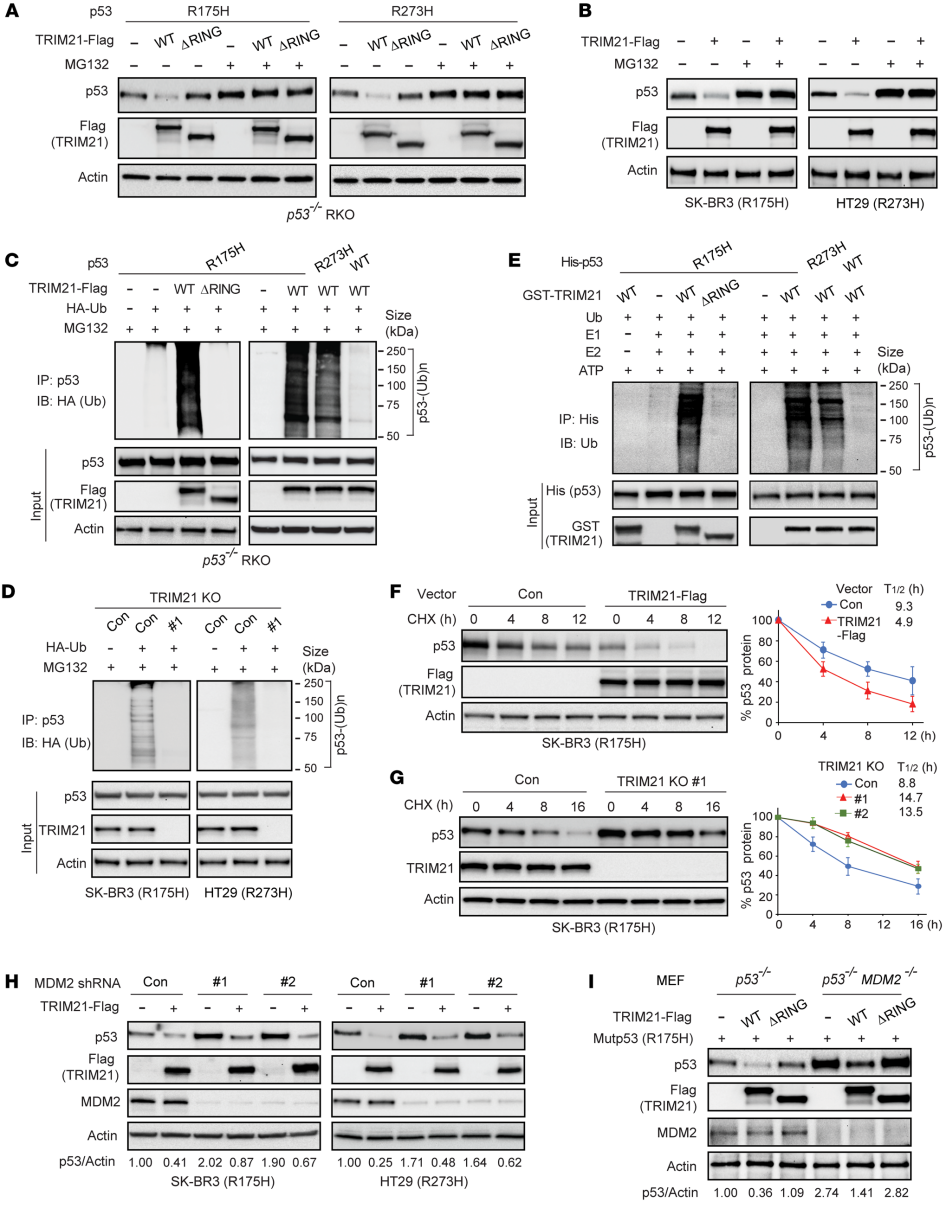
TRIM21 promotes mutp53 protein degradation through ubiquitination. (**A**) Ectopic expression of WT but not ΔRING TRIM21-Flag promoted the degradation of ectopic R175H and R273H mutp53 in *p53^−/−^* RKO cells, which was largely blocked by the proteasome inhibitor MG132. (**B**) MG132 treatment increased endogenous mutp53 protein levels and largely abolished the inhibitory effect of TRIM21-Flag on mutp53 protein levels in SK-BR3 and HT29 cells. (**C**) Ectopic expression of WT but not ΔRING TRIM21-Flag promoted ubiquitination of ectopic R175H and R273H mutp53 (but not wtp53) in *p53^−/−^* RKO cells analyzed by in vivo ubiquitination assays. Ub, ubiquitin. (**D**) TRIM21 KO reduced ubiquitination of endogenous R175H and R273H mutp53 in SK-BR3 and HT29 cells, respectively, analyzed by in vivo ubiquitination assays. (**E**) GST-TRIM21 protein ubiquitinated R175H and R273H His-mutp53 (but not His-wtp53) analyzed by in vitro ubiquitination assays using purified recombinant proteins. (**F**) Ectopic TRIM21-Flag expression decreased endogenous R175H mutp53 protein half-life in SK-BR3 cells. (**G**) TRIM21 KO increased endogenous R175H mutp53 protein half-life in SK-BR3 cells. In **F** and **G**, cells were treated with cyclohexamide (CHX) (50 μg/mL) for different amounts of time (hours) before Western blot assays. *n* = 3. (**H**) Ectopic TRIM21-Flag expression decreased mutp53 protein levels in both SK-BR3 and HT29 cells with or without MDM2 knockdown by 2 different shRNA vectors. (**I**) Ectopic expression of WT but not the ΔRING TRIM21-Flag decreased the levels of ectopic R175H mutp53 protein in both *p53^−/−^* and *p53^−/−^ MDM2^−/−^* MEFs.

We further investigated whether TRIM21 downregulates mutp53 protein levels through ubiquitin-mediated proteasomal degradation. The RING finger domain of E3 ubiquitin ligases is critical for their E3 ubiquitin ligase activity (23, 29, 46). Here, *p53^−/−^* RKO cells were cotransfected with vectors expressing R175H or R273H mutp53 together with vectors expressing the WT TRIM21-Flag or mutant TRIM21-Flag with deletion of the RING domain (ΔRING). The WT but not ΔRING TRIM21-Flag clearly downregulated the exogenous mutp53 protein levels in *p53^−/−^* RKO cells, which was abolished by treating cells with the proteasome inhibitor MG132 (Figure 3A). Similar results were observed in SK-BR3 and HT29 cells; the downregulation of endogenous R175H and R273H mutp53 by TRIM21-Flag was abolished by MG132 (Figure 3B). To investigate whether TRIM21 ubiquitinates mutp53, in vivo ubiquitination assays were performed in different cells. Ectopic expression of WT but not ΔRING TRIM21-Flag dramatically promoted ubiquitination of exogenous R175H and R273H mutp53 in *p53^−/−^* RKO cells (Figure 3C). In contrast, TRIM21-Flag displayed a very limited effect on wtp53 ubiquitination in cells (Figure 3C). Furthermore, TRIM21 KO decreased ubiquitination of endogenous R175H and R273H mutp53 in SK-BR3 and HT29 cells, respectively (Figure 3D). The TRIM21-mediated mutp53 ubiquitination was further confirmed by in vitro ubiquitination assays using recombinant proteins; WT GST-TRIM21 induced the ubiquitination of His-mutp53 (both R175H and R273H) but not His-wtp53, which was abolished by deletion of the TRIM21 RING domain (Figure 3E).

To test whether TRIM21 destabilizes mutp53, the protein half-life of mutp53 was analyzed. SK-BR3 cells with TRIM21-Flag expression or TRIM21 KO and their control cells were treated with the protein synthesis inhibitor cyclohexamide for different time periods before Western blot analysis of endogenous R175H mutp53 protein levels. The half-life of R175H mutp53 protein was clearly reduced by ectopic expression of TRIM21-Flag (Figure 3F) and increased by TRIM21 KO in cells (Figure 3G). Collectively, these results suggest that TRIM21 directly binds to mutp53 to ubiquitinate and degrade mutp53, and TRIM21 deficiency leads to mutp53 stabilization and accumulation.

MDM2 is the most critical negative regulator of wtp53, which ubiquitinates and degrades wtp53 (47, 48). MDM2 is frequently amplified and/or overexpressed in different types of cancers to inhibit p53 function in tumor suppression (8, 47, 48). MDM2 overexpression is often mutually exclusive with p53 mutations in many human cancers, supporting the important role of MDM2 in promoting tumorigenesis through its negative regulation of wtp53 (8, 47, 48). In addition to wtp53, MDM2 can also ubiquitinate and degrade mutp53 and mediate functions of some other mutp53 regulators, such as HSP90 and BAG2 (22-26, 40). To determine whether mutp53 degradation by TRIM21 is MDM2-dependent, MDM2 was knocked down by shRNA in SK-BR3 and HT29 cells. While MDM2 knockdown increased mutp53 protein levels, ectopic TRIM21-Flag expression efficiently decreased mutp53 protein levels in both cells with and without MDM2 knockdown, suggesting that TRIM21 downregulates mutp53 independently of MDM2 (Figure 3H). This result was confirmed in *p53^−/−^* and *p53^−/−^ MDM2^−/−^* MEFs; WT but not ΔRING TRIM21-Flag efficiently downregulated exogenous R175H mutp53 levels in both MEF lines (Figure 3I). Our results further showed that the role of MDM2 in downregulating mutp53 is also TRIM21 independent. TRIM21 KO did not clearly affect the inhibitory effect of ectopic MDM2-Flag on mutp53 protein levels in SK-BR3 and HT29 cells (Supplemental Figure 2A). Knockdown of MDM2 resulted in the increase of mutp53 protein levels in both WT and TRIM21 KO cells (Supplemental Figure 2B). Notably, while MDM2-Flag expression clearly reduced wtp53 protein levels in *p53^+/+^* MCF7 and RKO cells (Supplemental Figure 2C), TRIM21-Flag expression only slightly downregulated wtp53 protein levels in both cell lines (Figure 2A). Similarly, while MDM2 knockdown clearly increased wtp53 protein levels (Supplemental Figure 2D), TRIM21 knockdown only slightly induced wtp53 protein levels in cells (Figure 2C and Supplemental Figure 2, C and D). Furthermore, the inhibitory effect of MDM2 on wtp53 is TRIM21 independent; knockdown of TRIM21 did not clearly affect the inhibitory effect of MDM2 on wtp53 protein levels in cells (Supplemental Figure 2, C and D). These results suggest that both TRIM21 and MDM2 can independently downregulate mutp53 and, furthermore, unlike MDM2, TRIM21 does not play a critical role in negative regulation of wtp53 in cells.

### TRIM21 suppresses mutp53 accumulation to inhibit anchorage-independent growth of cancer cells.

**Figure 4:**
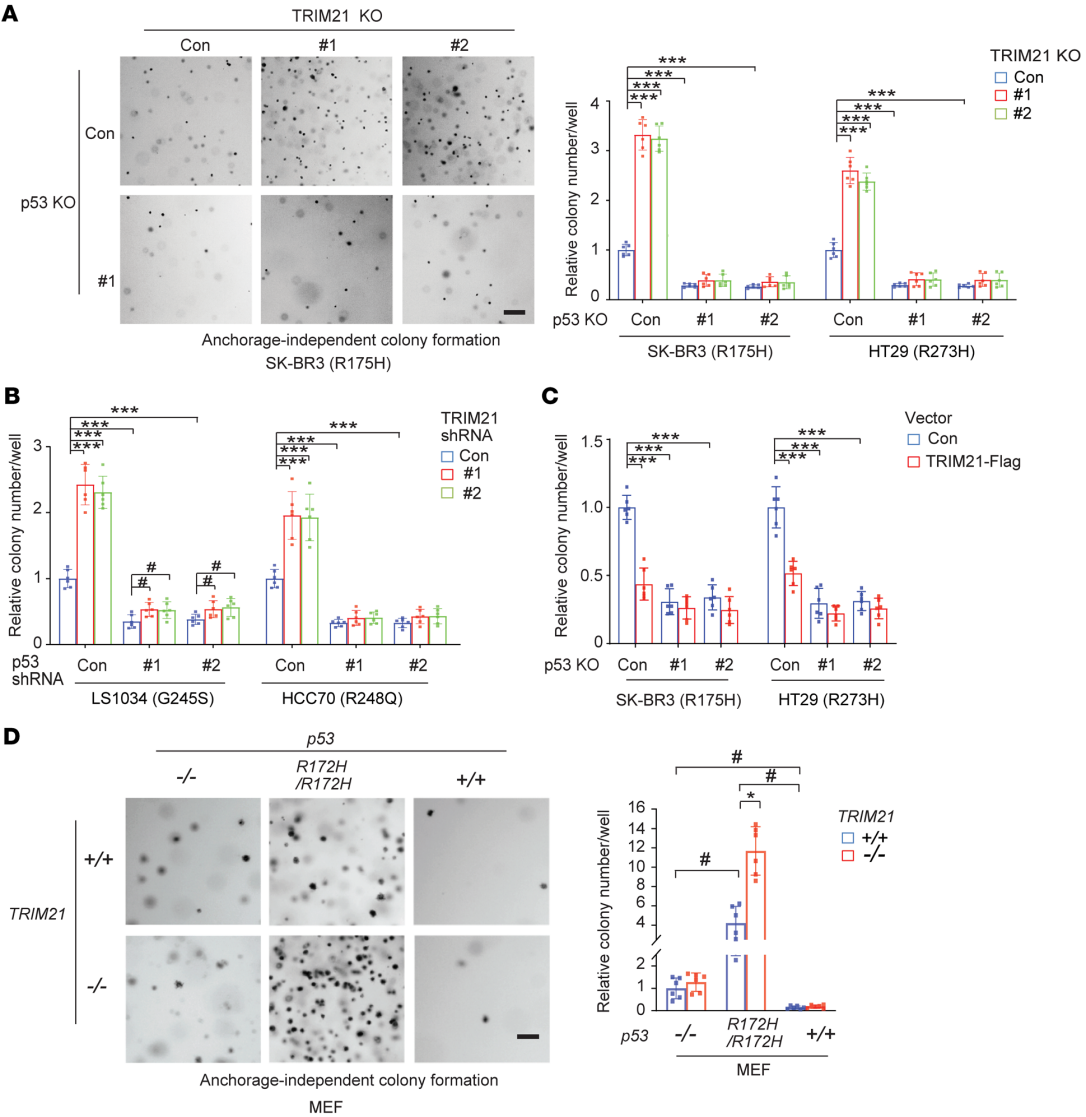
TRIM21 inhibits mutp53 GOF in promoting anchorage-independent growth of cancer cells. (**A**) TRIM21 KO enhanced anchorage-independent growth of SK-BR3 and HT29 cells, which was largely abolished by mutp53 KO. The control, TRIM21 KO, mutp53 KO, and TRIM21 + mutp53 double-KO cells were employed for anchorage-independent growth assays in soft agar. Left: Representative images of anchorage-independent growth of SK-BR3 cells. Right: Summary of relative cell colony numbers in soft agar. (**B**) Knockdown of endogenous TRIM21 by shRNA vectors enhanced anchorage-independent growth of LS1034 and HCC70 cells, which was largely abolished by mutp53 knockdown. The control, TRIM21 knockdown, mutp53 knockdown, and TRIM21 + mutp53 double-knockdown cells were employed for assays. (**C**) Ectopic TRIM21-Flag expression inhibited the anchorage-independent growth of SK-BR3 and HT29 cells, which was largely abolished by mutp53 KO. Control and mutp53 KO cells were transduced with control or TRIM21-Flag expression vectors for assays. (**D**) *TRIM21* loss enhanced mutp53 GOF activity in promoting anchorage-independent growth of E1A/RasV12-transformed MEFs. The E1A/RasV12-transformed *p53^R172H/R172H^*, *p53^−/−^*, and *p53^+/+^* MEFs with or without *TRIM21* loss (*TRIM21^−/−^* or *TRIM21^+/+^*) were employed for assays. Left: Representative images of anchorage-independent growth of MEFs. Data are shown as the mean ± SD (*n* = 6). ANOVA followed by Dunnett’s test. ^#^*P* < 0.05; *P < 0.01; ***P < 0.0001. Scale bars: 200 μm (**A** and **D**).

Given the critical role of mutp53 accumulation in mutp53 GOF, we determined the effect of TRIM21 on mutp53 GOF in cancer cells. Promoting cancer cell proliferation is a critical mutp53 GOF activity (6, 9, 10). It has been well known that wtp53 inhibits anchorage-independent growth of cancer cells in soft agar, whereas GOF mutp53 promotes anchorage-independent cell growth (49–52). Here, we investigated whether TRIM21 affects mutp53 GOF activity in promoting anchorage-independent cell growth. Anchorage-independent cell growth was significantly suppressed by KO of mutp53 in SK-BR3 and HT29 cells and knockdown of mutp53 in LS1034 and HCC70 cells, which demonstrated the GOF activities of these hot spot mutp53 in cancer cells (Figure 4, A and B). Notably, TRIM21 KO in SK-BR3 and HT29 cells significantly promoted anchorage-independent cell growth, which was largely abolished by mutp53 KO (Figure 4A). Consistently, TRIM21 knockdown significantly enhanced the anchorage-independent growth of LS1034 and HCC70 cells, which was largely abolished by mutp53 knockdown (Figure 4B). Furthermore, ectopic TRIM21-Flag expression significantly inhibited anchorage-independent growth of SK-BR3 and HT29 cells but displayed a much less inhibitory effect on their corresponding mutp53 KO cells (Figure 4C). Notably, TRIM21 knockdown displayed a much less pronounced promoting effect on anchorage-independent cell growth in both *p53^−/−^* and *p53^+/+^* RKO cells compared with these cell lines carrying mutp53 (Supplemental Figure 3). Consistent with previous studies (53, 54), loss of wtp53 in RKO cells promoted anchorage-independent cell growth (*p53^−/−^* vs. *p53^+/+^*), which demonstrates an inhibitory effect of wtp53 on anchorage-independent cell growth (Supplemental Figure 3). These results were further confirmed by employing E1A/RasV12-transformed *p53^+/+^*, *p53^−/−^*, and *p53^R172H/R172H^* MEFs with or without *TRIM21* deletion (*TRIM21^−/−^*) (Figure 4D). Anchorage-independent growth of transformed MEFs was significantly inhibited by wtp53 (*p53^+/+^* vs. *p53^−/−^*) but promoted by R172H mutp53 (*p53^R172H/R172H^* vs. *p53^−/−^*). Notably, *TRIM21* deletion significantly promoted anchorage-independent growth of *p53^R172H/R172H^* MEFs but not *p53^+/+^* or *p53^−/−^* MEFs (Figure 4D). These results together demonstrate that TRIM21 inhibits anchorage-independent cell growth through suppressing mutp53 accumulation and GOF.

### TRIM21 suppresses mutp53 accumulation to inhibit growth of orthotopic and s.c. tumors.

**Figure 5:**
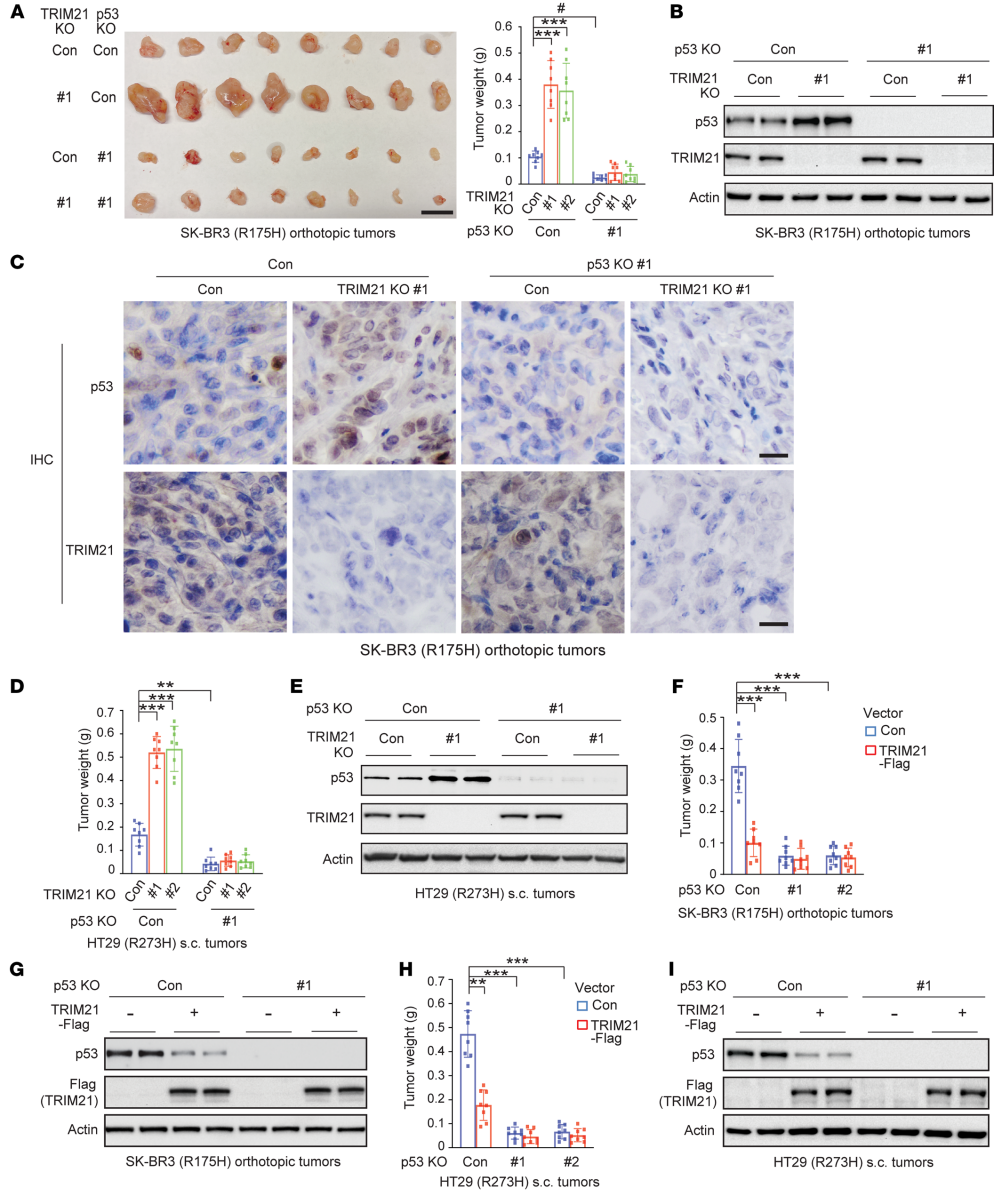
TRIM21 inhibits mutp53 GOF in promoting xenograft tumor growth. (**A**) TRIM21 KO in cells promoted the growth of orthotopic breast tumors formed by SK-BR3 cells, which was greatly abolished by mutp53 KO in cells. Left: Image of collected tumors. Right: The weights of collected tumors. Scale bar: 10 mm. *n* = 8 mice/group. (**B**) Western blot analysis of mutp53 and TRIM21 levels in SK-BR3 tumors described in **A**. (**C**) Representative images of IHC staining of mutp53 and TRIM21 in SK-BR3 tumors described in **A**. Scale bar: 20 μm. (**D**) TRIM21 KO in cells promoted the growth of s.c. xenograft tumors formed by HT29 cells, which was greatly abolished by mutp53 KO. *n* = 8 mice/group. (**E**) Western blot analysis of mutp53 and TRIM21 levels in HT29 tumors described in **D**. (**F**) TRIM21-Flag expression in cells inhibited the growth of SK-BR3 orthotopic breast tumors, which was greatly abolished by mutp53 KO. *n* = 8 mice/group. (**G**) Western blot analysis of mutp53 and TRIM21-Flag levels in SK-BR3 tumors described in **F**. (**H**) TRIM21-Flag expression in cells inhibited the growth of s.c. tumors formed by HT29 cells, which was greatly abolished by mutp53 KO. *n* = 8 mice/group. (**I**) Western blot analysis of p53 and TRIM21-Flag levels in HT29 tumors described in **H**. In **B**, **C**, **E**, **G**, and **I**, similar results were observed for 6 tumors/group that were analyzed, and representative results from 2 tumors/group (or 1 tumor/group in **C**) are presented. Data are shown as the mean ± SD. Two-way ANOVA followed by Dunnett’s or Bonferroni’s test. ^#^*P* < 0.05; **P < 0.001; ***P < 0.0001.

It has been well-established that wtp53 inhibits tumorigenesis whereas GOF mutp53 promotes tumorigenesis in xenograft tumor models (10, 13, 49, 50, 55). We further determined the effect of TRIM21 on mutp53 GOF in tumorigenesis by using orthotopic breast tumors formed by SK-BR3 cells and s.c. colorectal xenograft tumors formed by HT29 cells in athymic nude mice. Compared with control cells, TRIM21 KO in SK-BR3 cells significantly promoted the growth of SK-BR3 orthotopic tumors, whereas R175H mutp53 KO significantly inhibited tumor growth and largely abolished the promoting effect of TRIM21 KO on tumor growth (Figure 5A). Western blot analysis and IHC staining of SK-BR3 tumor tissues showed that TRIM21 KO clearly enhanced mutp53 protein levels (Figure 5, B and C) and tumor cell proliferation as reflected by the percentage of Ki-67–positive cells (Supplemental Figure 4A). Furthermore, mutp53 KO significantly reduced Ki-67–positive cell numbers and largely abolished the promoting effect of TRIM21 KO on Ki-67–positive cell numbers in SK-BR3 tumor tissues (Supplemental Figure 4A). Similar results were observed in HT29 s.c. tumors; while TRIM21 KO in HT29 cells significantly promoted tumor growth and clearly increased mutp53 protein levels in tumors, mutp53 KO in HT29 cells significantly inhibited tumor growth and largely abolished the promoting effect of TRIM21 KO on tumor growth (Figure 5, D and E, and Supplemental Figure 4B). Furthermore, TRIM21-Flag expression significantly inhibited the growth of SK-BR3 orthotopic tumors (Figure 5F and Supplemental Figure 4C) and HT29 s.c. tumors (Figure 5H), which was largely abolished by mutp53 KO in these cell lines (Figure 5, F and H, and Supplemental Figure 4C). Western blot analysis confirmed that TRIM21-Flag expression greatly reduced mutp53 levels in both SK-BR3 and HT29 tumors (Figure 5, G and I). It is worth noting that while s.c. xenograft tumors formed by *p53^−/−^* RKO cells grew much faster than tumors formed by *p53^+/+^* RKO cells, which indicates the tumor-suppressive function of wtp53, TRIM21 knockdown displayed a much less pronounced growth-promoting effect on *p53^+/+^* RKO tumors compared with its effect on the above-mentioned mutp53 tumors (Supplemental Figure 4D). Furthermore, TRIM21 knockdown displayed a similar mild promoting effect on both *p53^+/+^* and *p53^−/−^* RKO tumors, suggesting that this effect of TRIM21 knockdown on RKO tumors is largely wtp53 independent (Supplemental Figure 4D). These results together demonstrate that TRIM21 suppresses mutp53 accumulation to inhibit mutp53 GOF in promoting tumor growth.

### Low TRIM21 expression in cancers carrying mutp53 is associated with poor prognosis of patients with cancer.

**Figure 6:**
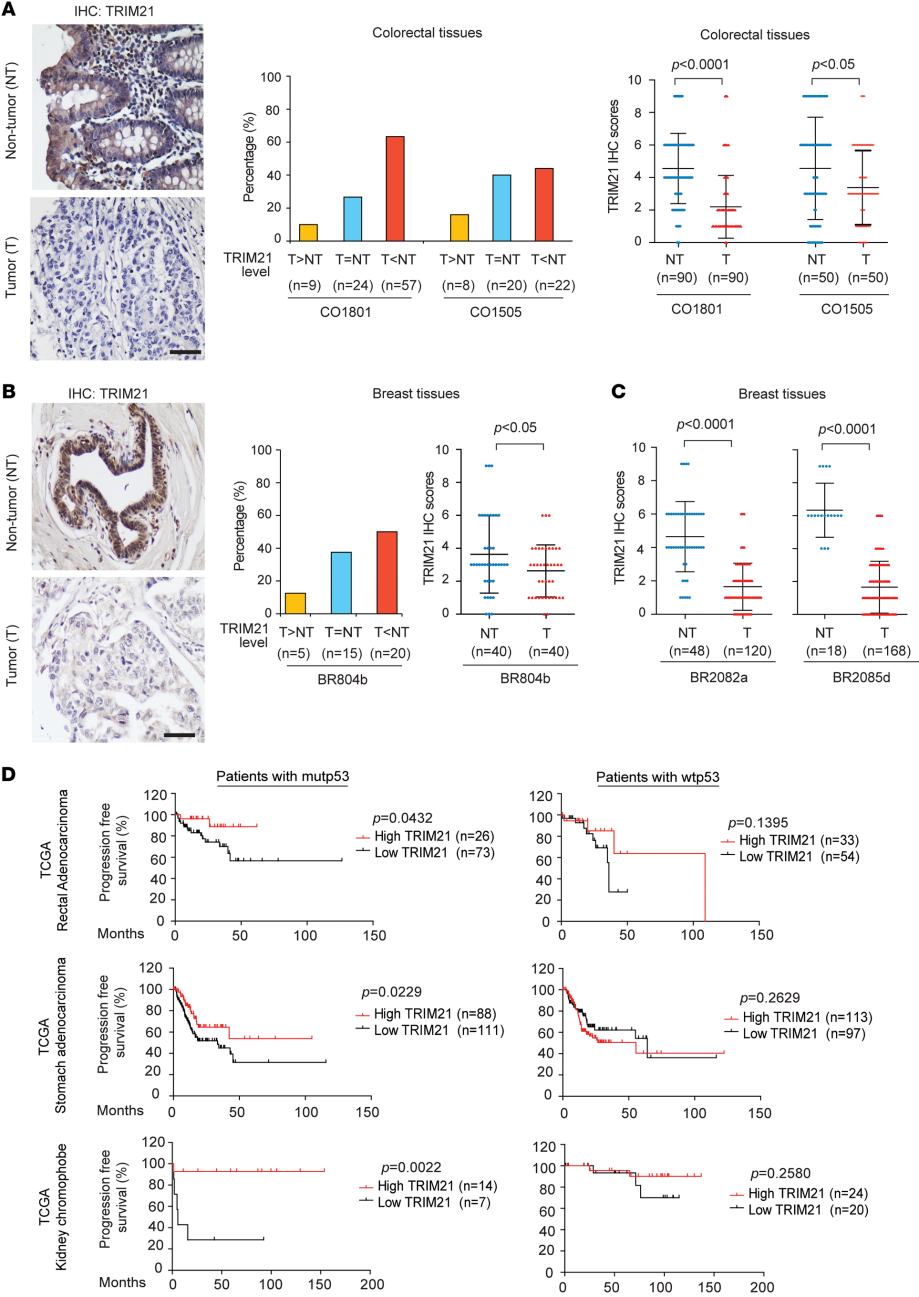
Decreased TRIM21 expression in human cancers and low TRIM21 expression is correlated with poor prognosis in patients with mutp53 cancers. (**A** and **B**) TRIM21 protein levels were frequently decreased in 2 cohorts of colorectal tumor specimens (**A**) and a cohort of breast tumor specimens (**B**) compared with their matched adjacent nontumor tissues in TMAs, as analyzed by IHC staining. Left: Representative IHC staining images of TRIM21 in tumors (T) and their matched adjacent nontumor tissues (NT). Scale bar: 60 μm. Middle: TRIM21 protein levels were decreased in a high percentage of colorectal and breast tumor specimens. Right: TRIM21 protein levels were significantly decreased in colorectal and breast tumor specimens. (**C**) TRIM21 protein levels were significantly decreased in two additional cohorts of breast tumor specimens compared with nontumor breast tissues in TMAs, as analyzed by IHC staining. In **A**–**C**, TMAs were obtained from US Biomax. IHC scores were obtained as described in Methods. Data are shown as the mean ± SD. Statistical analysis was performed using 2-tailed unpaired Student’s *t* test. (**D**) Low *TRIM21* mRNA expression is associated with poor progression-free survival in patients with mutp53 cancers. Kaplan-Meier survival analysis was employed. The survival information and *TRIM21* expression *z* score relative to normal samples were obtained from cBioPortal. The patients were divided into low and high *TRIM21* expression groups according to the cut-off of *z* score = 0. The difference between the two survival curves was analyzed using the log-rank (Mantel-Cox) test.

TRIM21 expression was reported to be frequently decreased in some types of cancers, including colorectal and breast cancers (35–37). Consistently, we found that TRIM21 protein levels were frequently downregulated in colorectal and breast cancers by IHC staining analysis of TRIM21 expression in multiple cohorts of colorectal and breast cancers using tissue microarrays (TMAs; obtained from US Biomax) (Figure 6, A and B). Compared with matched adjacent nontumor samples, TRIM21 protein levels were downregulated in 63% (*n* = 57 of 90) and 44% (*n* = 22 of 50) of two different cohorts of tumor samples, respectively (Figure 6A), and in 50% (*n* = 20 of 40) of a cohort of colorectal cancer samples (Figure 6B). Furthermore, compared with their matched adjacent nontumor tissues, the average protein levels of TRIM21 in colorectal and breast cancers were significantly decreased (Figure 6, A and B). These results were confirmed in an additional 2 cohorts of breast cancer samples using TMAs; compared with nontumor breast tissues, TRIM21 protein levels were significantly downregulated in these breast cancers (Figure 6C). In addition to the decreased expression of TRIM21 in colorectal and breast tumors, the alteration of TRIM21 expression was also observed in some other types of tumors. Analysis of The Cancer Genome Atlas (https://portal.gdc.cancer.gov/) database showed that, compared with matched nontumor tissues, downregulation of TRIM21 mRNA was frequently observed in lung cancer (60 of 109), and overexpression of TRIM21 mRNA was frequently observed in kidney clear cell carcinoma (49 of 72), stomach cancer (11 of 32), and head and neck squamous cell cancer (14 of 43) (Supplemental Figure 5). Interestingly, analysis of TRIM21 expression in The Cancer Genome Atlas database from cBioPortal (https://www.cbioportal.org/) showed that low TRIM21 expression in several types of tumors was significantly associated with poor clinical outcomes in patients with mutp53 cancers but not wtp53 cancers, including rectal adenocarcinoma, stomach adenocarcinoma, and kidney chromophobe (Figure 6D), as well as breast invasive ductal carcinoma (Supplemental Figure 6). These results suggest a tumor-suppressive function of TRIM21 in some cancers carrying mutp53.

### TRIM21 deletion results in mutp53 accumulation in normal tissues and promotes tumorigenesis in p53^R172H/R172H^ mice.

R172H mutp53–knockin mice have been widely used for mutp53 GOF studies (41–43). It has been reported that mutp53 specifically accumulated in tumors but not normal tissues in mutp53-knockin mice, including R172H mutp53–knockin mice, indicating that mutp53 accumulation is a tumor-specific event (41, 42). Furthermore, *MDM2* loss in R172H mutp53–knockin mice resulted in mutp53 accumulation in normal tissues, an earlier tumor onset and reduced life span of mice, indicating that mutp53 accumulation is required for mutp53 GOF in tumorigenesis (24).

**Figure 7:**
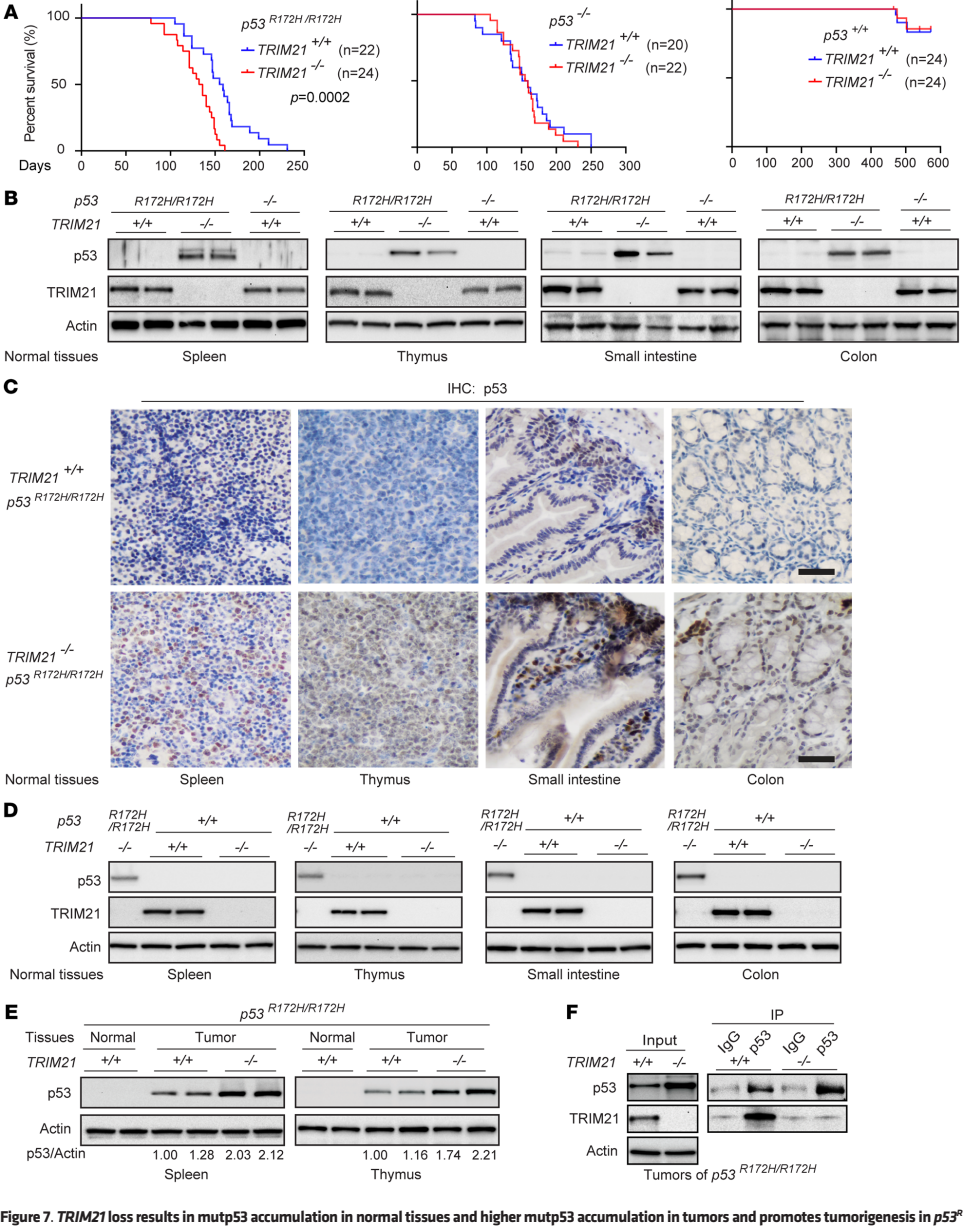
*TRIM21* loss results in mutp53 accumulation in normal tissues and higher mutp53 accumulation in tumors and promotes tumorigenesis in *p53^R172H/R172H^* mice. (**A**) The effect of *TRIM21* loss on the survival of *p53^R172H/R172H^* (left), *p53^−/−^* (middle), and *p53^+/+^* mice (right). Kaplan-Meier survival curves are presented and the difference in survival was analyzed by the log-rank (Mantel-Cox) test. (**B** and **C**) R172H mutp53 protein accumulation in different normal tissues of 4-week-old *TRIM21^−/−^ p53^R172H/R172H^* mice but not *TRIM21^+/+^ p53^R172H/R172H^* mice analyzed by Western blot analysis (**B**) and IHC staining of mutp53 (**C**). Scale bar: 20 μm. (**D**) *TRIM21* loss did not result in wtp53 protein accumulation in normal tissues of p53^+/+^ mice analyzed by Western blot assays. Different normal tissues from 4-week-old *TRIM21^+/+^ p53^+/+^* and *TRIM21^−/−^ p53^+/+^* mice as well as *TRIM21^−/−^ p53^R172H/R172H^* mice (as positive controls) were employed for analysis. (**E**) Tumors (splenic and thymic lymphomas) of *TRIM21^−/−^ p53^R172H/R172H^* mice displayed higher mutp53 protein accumulation than tumors of *TRIM21^+/+^ p53^R172H/R172H^* mice. In **B**–**E**, similar results were observed in samples from at least 5–8 mice/group, and results from 2 mice/group are presented. (**F**) The interaction between TRIM21 and mutp53 proteins in thymic lymphomas of *TRIM21^+/+^ p53^R172H/R172H^* and *TRIM21^−/−^ p53^R172H/R172H^* mice analyzed by co-IP assays.

To determine the effect of TRIM21 on mutp53 accumulation and GOF in vivo, we employed *TRIM21^+/+^ p53^R172H/R172H^* and *TRIM21^−/−^ p53^R172H/R172H^* mice. It has been reported that the *TRIM21^−/−^* mice used in this study are fertile and have a normal life span (56). Intriguingly, while *TRIM21* loss did not clearly affect the life span of *p53^−/−^* or *p53^+/+^* mice, *TRIM21* loss resulted in an earlier tumor onset and significantly reduced life span of *p53^R172H/R172H^* mice. *TRIM21^+/+^ p53^R172H/R172H^* and *TRIM21^−/−^ p53^R172H/R172H^* mice had a median survival of 157 and 134 days, respectively (*P* = 0.0002; Figure 7A). Furthermore, both *TRIM21^+/+^ p53^R172H/R172H^* and *TRIM21^−/−^ p53^R172H/R172H^* mice developed mostly lymphomas and sarcomas, and no significant difference in the tumor spectrum was observed (Supplemental Table 2). Notably, while mutp53 accumulation was not observed in normal tissues of *p53^R172H/R172H^* mice, including the spleen, thymus, small intestine, and colon tissues, mutp53 protein accumulated to high levels in these normal tissues in *TRIM21^−/−^ p53^R172H/R172H^* mice, as analyzed by both Western blot and IHC analysis (Figure 7, B and C). It has been well-established that wtp53 protein levels are kept at very low levels in normal tissues under normal (nonstress) conditions (Figure 7D), which are maintained through MDM2-mediated ubiquitination and degradation of wtp53 (47). *MDM2* KO in mice resulted in embryonic lethality due to uncontrolled wtp53 accumulation and activation and the resultant wtp53-mediated apoptosis, which can be rescued by p53 KO in mice (57). In addition, loss of *MDM2* in adult mice caused drastic wtp53 accumulation and activation in various normal tissues, leading to lethality of adult mice (58). These in vivo results clearly demonstrated the vital role of MDM2 in negative regulation of wtp53. Interestingly, unlike *MDM2* loss, *TRIM21* loss did not result in wtp53 protein accumulation in normal tissues in *p53^+/+^* mice, indicating that TRIM21 does not play an important role in wtp53 regulation in vivo under normal conditions (Figure 7D). Furthermore, while mutp53 accumulated in tumors of both *TRIM21^+/+^ p53^R172H/R172H^* and *TRIM21^−/−^ p53^R172H/R172H^* mice, higher levels of mutp53 accumulation were observed in *TRIM21^−/−^ p53^R172H/R172H^* tumors compared with *TRIM21^+/+^ p53^R172H/R172H^* tumors (Figure 7E). Results from real-time PCR assays showed that *TRIM21* loss did not affect *mutp53* mRNA levels in normal or tumor tissues in *p53^R172H/R172H^* mice (Supplemental Figure 7). The interaction between TRIM21 and R172H mutp53 proteins in tumors was confirmed by co-IP assays, indicating that TRIM21 interacts with mutp53 in vivo (Figure 7F). Taken together, these results demonstrate that *TRIM21* loss results in mutp53 accumulation in normal tissues and further accumulation of mutp53 in tumors, leading to an earlier tumor onset and reduced life span of *p53^R172H/R172H^* mice.

## Discussion

**Figure 8:**
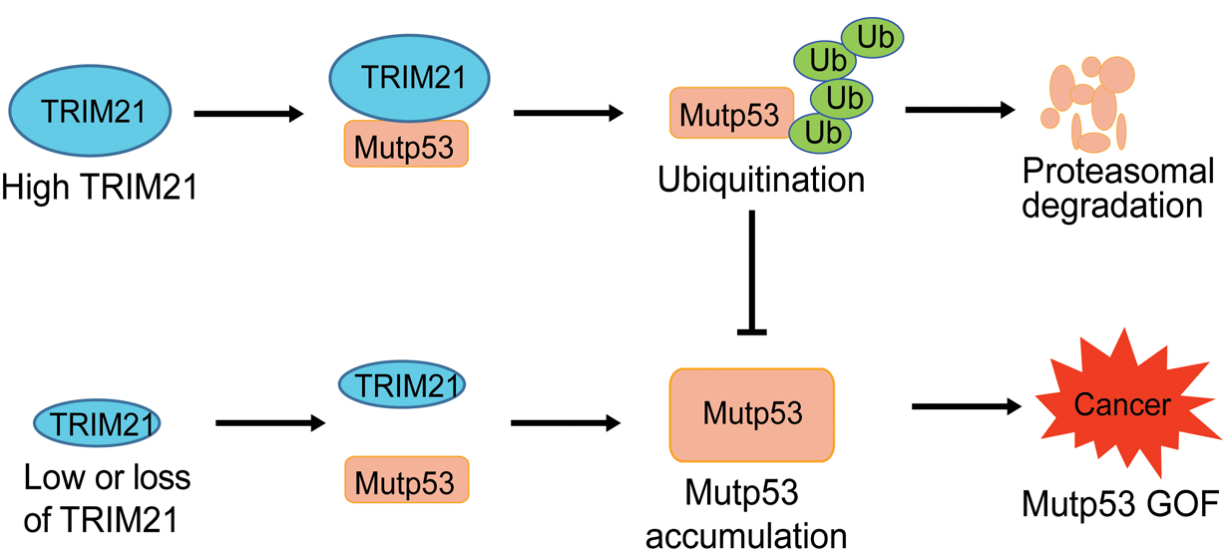
The schematic model depicting the regulation of mutp53 by TRIM21. A schematic model showing that TRIM21 interacts with mutp53 and ubiquitinates and degrades mutp53 to suppress mutp53 GOF in tumorigenesis, and TRIM21 deficiency results in mutp53 stabilization and accumulation in cancer cells to promote tumorigenesis.

mutp53 accumulation in tumors is crucial for mutp53 GOF in tumorigenesis (6, 7, 9, 10, 12, 22–25). Currently, the mechanism for mutp53 accumulation in tumors is not well-understood. It had been widely accepted that MDM2 cannot effectively ubiquitinate and degrade mutp53, resulting in mutp53 accumulation in tumors. However, studies of GOF mutp53-knockin mice, including R172H mutp53–knockin mice, showed that mutp53 accumulated specifically in tumors but not normal tissues, indicating that MDM2 can effectively degrade mutp53 in normal tissues but not in tumor tissues (41, 42), Furthermore, *MDM2* deletion in R172H mutp53–knockin mice resulted in mutp53 accumulation in normal tissues, which in turn promoted tumor development and reduced mouse life span (24). These results from genetically engineered mouse models, together with other results from cell cultures, have demonstrated that while MDM2 can effectively degrade mutp53 in cultured normal and cancer cells as well as normal mouse tissues, tumors develop specific mechanisms to impair MDM2-mediated mutp53 degradation, leading to mutp53 accumulation in tumors (7, 24–26, 59). Some of these tumor-specific mechanisms have been reported. For instance, HSP90 binds to mutp53 and inhibits MDM2-mediated mutp53 degradation, leading to mutp53 accumulation (22, 60). Targeting HSP90 reactivates MDM2 function in degrading mutp53, which destabilizes mutp53 to suppress mutp53 GOF in tumorigenesis (22). Our previous studies showed that increased expression of tumor-associated MDM2 short isoforms (e.g., the isoform B) and BAG2 in tumors inhibits MDM2-mediated mutp53 degradation, leading to mutp53 accumulation in tumors to promote tumorigenesis (25, 40). The *INK4a-ARF* locus encodes 2 proteins, p16^INK4a^ and p14^ARF^ (murine p19^ARF^), that function in tumor suppression. p16^INK4a^ inhibits the activity of CDK4 and CDK6, and p14^ARF^ binds to and inhibits MDM2 to activate p53 (61). *p16^INK4a^* loss, frequently observed in human cancers, can activate p14^ARF^, which in turn activates wtp53. Like *MDM2* deletion, *p16 ^INK4a^* deletion in mice leads to mutp53 accumulation in normal tissues and promotes mutp53 GOF in tumorigenesis (24). In addition, multiple cancer-related stress stimuli, including DNA damage, oxidative and proteotoxic stress, and metabolic stress, have been reported to promote mutp53 protein accumulation and GOF in tumors through different mechanisms (12, 62, 63). Although these studies revealed some important mechanisms underlying mutp53 accumulation in human cancer, the precise mechanism of mutp53 accumulation is far from clear. In this study, we identified TRIM21 as a specific mutp53-binding protein; TRIM21 directly binds to mutp53 (but not wtp53), resulting in the ubiquitination and degradation of mutp53 to suppress mutp53 GOF in tumorigenesis. Furthermore, TRIM21 degrades mutp53 independently of MDM2, and similarly, MDM2 degrades mutp53 independently of TRIM21. Like *MDM2* deletion, *TRIM21* deletion in mice leads to mutp53 accumulation in normal tissues, promoting tumor development and reducing the life span of mice. Thus, our results strongly suggest that the ubiquitination of mutp53 by TRIM21 is a critical mechanism underlying mutp53 regulation in cancer cells, and furthermore, the downregulation of TRIM21 expression, which is frequently observed in some cancers, including colorectal and breast cancers, is an important mechanism contributing to mutp53 accumulation and GOF in cancer (Figure 8).

Interestingly, both tumor-suppressive and oncogenic roles of TRIM21 have been reported, and mechanisms of these seemingly contradictory roles of TRIM21 in cancer remain elusive (35–39). In addition to our finding that TRIM21 directly regulates mutp53, TRIM21 was reported to indirectly downregulate wtp53 protein levels through ubiquitinating GMPS to inhibit wtp53 deubiquitination by USP7 in response to DNA damage (34, 64). Here, we found that compared with mutp53, the inhibitory effect of TRIM21 on wtp53 was much less pronounced or minimal in different cells, including MCF7, *p53^+/+^* RKO, and *p53^+/+^* MEF cells. Importantly, while *TRIM21* loss resulted in mutp53 accumulation in normal tissues, an earlier tumor onset, and shortened life span in *mutp53^R172H/R172H^* mice, *TRIM21* loss did not result in wtp53 accumulation in normal tissues or affect tumorigenesis and life span in *p53^+/+^* mice. These results suggest that while TRIM21 is involved in the fine-tuning of wtp53 levels in response to DNA damage, TRIM21 plays an important role in regulating mutp53 levels and GOF in cancer and TRIM21 deficiency promotes mutp53 accumulation and cancer progression. These results reveal that negative regulation of mutp53 is an important mechanism for TRIM21 in tumor suppression, and at the same time, also suggest that as an E3 ubiquitin ligase, the precise role of TRIM21 in tumor suppression or promotion may depend on its direct major protein substrates in different types of cells and tissues, which express different levels of TRIM21 substrates. Therefore, future studies are needed to better characterize TRIM21 substrates and their contributions to tumorigenesis. In addition, currently, it remains unclear why TRIM21 specifically binds to mutp53 but not wtp53 and why TRIM21 appears to show a stronger interaction with R175H than other hot spot mutp53 that we tested, which deserve future studies.

The emerging critical role of TRIM family proteins, including TRIM21, in human cancers suggests the potential application of these proteins in cancer therapies (29–31). For instance, given that TRIM21 negatively regulates mutp53 GOF and TRIM21 expression is frequently downregulated in some cancer types, enhancing the protein levels and/or E3 ubiquitin ligase activity of TRIM21 might provide a therapeutic strategy for some cancers carrying GOF mutp53. However, further studies are required to reveal the precise regulation of TRIM21 levels and activity in normal and cancer cells and tissues. Like TRIM21, the role and mechanism of many other TRIM family proteins in cancer were reported to be highly cancer type and context dependent, suggesting that many more studies are urgently needed to determine the precise role and mechanism of TRIM family proteins in different cancer types and under different circumstances before feasible therapeutic strategies can be developed to target TRIM family proteins in cancer.

In summary, our results revealed a critical mechanism for mutp53 accumulation and GOF in cancer and also identified an important mechanism underlying the tumor-suppressive function of TRIM21 in cancer. Better understanding of the mechanism of mutp53 accumulation and GOF in cancer will provide new opportunities to develop effective therapeutic strategies for cancers carrying mutp53.

## Methods

### Cell cultures and vector constructs.

SK-BR3, HT29, HCC70, LS1034, and MCF7 cell lines were obtained from ATCC. *p53^+/+^* RKO and its isogenic *p53^−/−^* RKO cell lines were gifts from Bert Vogelstein (Johns Hopkins University, Baltimore, Maryland, USA). *p53^−/−^* and *p53^−/−^ MDM2^−/−^* MEFs were gifts from Guillermina Lozano (MD Anderson Cancer Center, Houston, Texas, USA). Other MEFs were isolated from 13.5 day embryos of genetically modified mice according to the standard procedures (65). The pLPCX vectors expressing mutp53, TRIM21, and their deletion mutants were constructed by PCR amplification. Two shRNA vectors against TRIM21 were constructed by inserting the following sequences for human TRIM21 siRNA into the PLKO.1 puro lentiviral shRNA vector (Addgene, 8453): TRIM21 no. 1, 5′-UCAUUGUCAAGCGUGCUGC-3′ and TRIM21 no. 2, 5′-UGGCAUGGAGGCACCUGAAGGUGG-3′. Two shRNA vectors against p53 were constructed by inserting the following sequences for human p53 siRNA into the PLKO.1 hygro lentiviral shRNA vector (Addgene, 24150): p53 no. 1, 5′-GACUCCAGUGGUAAUCUACU-3′ and p53 no. 2, 5′-GUCCAGAUGAAGCUCCCAGAA-3′. Two lentiviral shRNA vectors against MDM2 (V2LHS_151657 and V3LHS_379468) were obtained from Open Biosystems.

### Generation of TRIM21 and mutp53 KO cell lines using CRISPR/Cas9.

The single guide RNAs (sgRNAs) were designed by the CRISPR sgRNA design web tool as described previously (65). The sgRNA sequences were as follows: mutp53, sgRNA-a, 5′-CCATTGTTCAATATCGTCCG-3′, and sgRNA-b, 5′-GGGCAGCTACGGTTTCCGTC-3′; TRIM21, sgRNA-a, 5′-ATGCTCACAGGCTCCACGAA-3′, and sgRNA-b, 5′-TCATCTCAGAGCTAGATCGA-3′. The annealed oligonucleotides were ligated into the pSpCas9n(BB)-2A-GFP vector (Addgene, 48140). KO lines were generated as we previously described (65). In brief, cells transfected with 2 sgRNAs were sorted by flow cytometry, and the GFP-positive single cells were seeded. Single-cell colonies were selected by sequencing PCR products of the edited regions. The deletion of TRIM21 and mutp53 was validated by Western blot assays.

### LC-MS/MS assays.

To determine potential mutp53-binding proteins, *p53^−/−^* RKO cells transduced with the empty control vector or vectors expressing R175H mutp53 or wtp53 were employed for co-IP by using the anti-p53 (DO-1) beads (Santa Cruz Biotechnology, sc-126AC). The p53 protein complex was eluted with 0.1 M glycine solution, separated in a SDS-PAGE gel, visualized by silver staining using a silver staining kit (Invitrogen), and analyzed by LC-MS/MS at the Biological Mass Spectrometry facility of Rutgers University.

### Western blot assays.

Standard Western blot assays were used to analyze protein expression. The following antibodies were used for assays: anti-Flag-M2 (Sigma-Aldrich, F1804), anti–β-Actin (Sigma-Aldrich, A5441), anti-HA (Roche, 3F10), anti-His (Santa Cruz Biotechnology, sc-803), anti-GST (Santa Cruz Biotechnology, sc-138), anti-human p53 (Santa Cruz Biotechnology, sc-126), anti-TRIM21 (Abcam, ab207728), anti-MDM2 (2A-10) (66), anti-Ub (Santa Cruz Biotechnology, sc-8017), and anti-p53 (Leica Biosystems, CM5). All intensity quantification for Western blot was performed by using ImageJ software (NIH).

### In vitro GST pull-down assays.

In vitro GST pull-down assays were performed as we previously described (65). In brief, *E*. *coli* (BL21 DE3 strain) transformed with GST-TRIM21 or His-mutp53 (R175H) vectors was induced with 0.4 mM IPTG for 16 hours at 16°C to express GST-TRIM21 or His-mutp53 (R175H) proteins. The purified GST-TRIM21 proteins were immobilized on Glutathione-Sepharose beads (GE Healthcare, 17-0756-01), which were then incubated with purified His-mutp53 (R175H) or His-wtp53 proteins. GST protein alone was used as a negative control. After washing, proteins bound to the beads were analyzed by Western blot assays using an anti-His (Santa Cruz Biotechnology, sc-803) or anti-GST antibody (Santa Cruz Biotechnology, sc-138).

### Analysis of gene expression.

The expression of genes in cells and tissues was analyzed by quantitative TaqMan real-time PCR assays. Total RNA in cells and tissues was prepared with the RNeasy kit (QIAGEN). The cDNA was prepared using a TaqMan Reverse Transcription kit (Applied Biosystems), and the real-time PCR assay was performed using the TaqMan PCR Mixture (Applied Biosystems) according to the manufacturer’s instructions (67). All primers were obtained from Applied Biosystems. The expression of genes was normalized to *Actin*.

### In vivo and in vitro ubiquitination assays.

In vivo ubiquitination assays were performed as described previously (65, 68). In brief, cells were transfected with different expression vectors, including TRIM21-Flag, mutp53 or wtp53, and HA-Ub. Cells were then treated with 10 μM MG132 for 8 hours before cells were collected for assays. The levels of mutp53 ubiquitination in cells were determined by immunoprecipitation with an anti-p53 antibody (Santa Cruz Biotechnology, sc-126) followed by Western blot analysis with an anti-HA antibody (Sigma-Aldrich, 11867423001).

In vitro ubiquitination assays were performed as previously described (65, 68). In brief, the reaction mixtures (50 μL) contained 5 mM MgCl_2_, 50 mM Tris (pH 7.4), 1 mM DTT, 2 mM ATP, E1 (0.5 μg, Boston Biochem), E2 (0.5 μg; Boston Biochem), Ub (5 μg, Boston Biochem), purified recombinant His-mutp53 or His-wtp53 protein (0.5 μg), and purified recombinant GST-TRIM21 (0.5 μg). After incubation for 3 hours at 37°C, the mixtures were subjected to Western blot assays using an anti-Ub antibody (Santa Cruz Biotechnology, sc-8107) to measure the levels of mutp53 ubiquitination in vitro.

### Analysis of the half-life of mutp53 proteins.

The half-life of mutp53 proteins in cells were determined as previously described (65). Briefly, cells were treated with protein synthesis inhibitor cyclohexamide (50 μg/mL) or DMSO for indicated time periods (4–16 hours) before being collected for Western blot analysis of mutp53 protein levels using the anti-p53 antibody (Santa Cruz Biotechnology, sc-126).

### Anchorage-independent growth assays.

Anchorage-independent growth assays were performed as previously described (67). In brief, cells were seeded in 6-well culture plates coated with media containing 0.6% agarose and cultured in media containing 0.3% agarose. To establish E1A/RasV12-transformed *p53^+/+^*, *p53^−/−^,* and *p53^R172H/R172H^* MEFs with or without *TRIM21* deletion (*TRIM21^−/−^*), MEFs derived from mice were transduced with pBabe-E1A and pBabe-RasV12 retroviral vectors (Addgene). Equal amounts of infected cells (4 × 10^4^/well) were plated in soft agar in 6-well plates. Colonies were stained by crystal violet for counting after 2 weeks.

### Mice and mouse experiments.

*p53^−/−^* mice (no. 002101) were obtained from The Jackson Laboratory. *p53^R172H/R172H^* mice were a gift from Guillermina Lozano (42). *TRIM21^−/−^* mice were a gift from Keiko Ozato (NIH) (56, 69). *p53^−/−^* and *p53^R172H/R172H^* mice were crossed with *TRIM21^−/−^* mice to generate *TRIM21^−/−^ p53^−/−^* and *TRIM21^−/−^ p53^R172H/R172H^* mice, respectively.

For orthotropic breast tumor models, SK-BR3 cells (5 × 10^6^ cells in a 50:50 mix of DMEM/Matrigel) were injected into the mammary fat pads of 8-week-old female BALB/c athymic nude mice (Taconic; *n* = 8 female mice/group) as described previously (65). For s.c. xenograft tumor models, HT29, *p53^+/+^* RKO, and *p53^−/−^* RKO cells (5 × 10^6^ in 0.2 mL of PBS) were injected s.c. into 8-week-old BALB/c athymic nude mice (Taconic; *n* = 8 mice/group; half male and half female) as described previously (65, 67). At the endpoint, mice were killed and tumors were collected. Tumor weights were measured, and tumors were subjected to subsequent analysis.

### IHC assays.

The human colorectal cancer (CO1801 and CO1505) and breast cancer TMAs (BR804b, BR2082a, and BR2085d) were obtained from US Biomax. All specimens were deidentified. The IHC staining was performed as we described previously (65, 67). The anti-TRIM21 (Proteintech, 12108-1-AP), anti-p53 (Leica Biosystems, CM5), and anti-Ki-67 (BD Biosciences, 556003) antibodies were used to detect the levels of TRIM21, mutp53, and Ki-67 in TMAs and sections of tumors from mice, respectively. The IHC results were scored by using a scoring system from 0 to 9 as previously described (65, 67). In brief, the scores were obtained by multiplying the intensity of signals with the percentage of positive cells (signal: 0, no signal; 1, weak signal; 2, intermediate signal; 3, strong signal; percentage: 0, 0%; 1, <25%; 2, 25%–50%; 3, >50%). For Ki-67 staining, sections of 6 different tumors were counted for each group, and 5 fields of view from each section were randomly selected and counted. The number of Ki-67–positive cells was divided by the total cell number, and the percentages for each group were averaged.

### Statistics.

All data were obtained from at least 3 repetitions and were expressed as mean ± SD, as indicated in the figure legends. Two-tailed Student’s *t* test was applied for statistical analysis between 2 groups. ANOVA followed by Dunnett’s or Bonferroni’s multiple comparison tests was applied for multiple-group comparisons. The survival information of patients and mice was summarized by the Kaplan-Meier plots, and the difference was analyzed by the log-rank (Mantel-Cox) test. The statistical analysis was performed using GraphPad Prism 9. *P* values of less than 0.05 were considered significant.

### Study approval.

All animal procedures were approved by the IACUC at Rutgers University and performed in accordance with the IACUC guidelines.

## Author contributions

Juan Liu, CZ, DX, TZ, CYC, JW, and Jie Liu performed the experiments and analyzed data. LZ, BGH, and WXZ analyzed data and contributed important materials. WH and ZF conceived and supervised the study. Juan Liu, WH, and ZF wrote the manuscript.

## Supplementary Material

Supplemental data

## Version 1 02/07/2023

In-Press Preview

## Version 2 03/15/2023

Electronic publication
