# Supplementary material for: The ubiquitin ligase TRIM21 regulates mutant p53 accumulation and gain of function in cancer
Source: J Clin Invest. 2023 Mar 15;133(6):e164354. doi: 10.1172/JCI164354 (PMC10014102; doi:10.1172/JCI164354)
Supplement: Supplemental data [file jci-133-164354-s176.pdf]

Supplemental Material:

Supplemental Table 1

Potential mutp53-interacting proteins identified by co-IP followed by LC-MS/MS analysis

Potential p53-interacting proteins	Number of peptides		
	Con	wtp53	mutp53 (R175H)
TP53	0	172	164
HSPA8	1	10	120
HSP1A1	0	0	79
HSP90AB1	0	0	34
CCT2	0	2	21
**TRIM21**	**1**	**1**	**15**
BAG2	0	1	9
MYH4	0	0	5

p53$^{-/-}$ RKO cells transduced with vectors expressing wtp53 or R175H mutp53 and control (Con) *p53*$^{-/-}$ RKO cells transduced with empty vectors were employed for analysis. The wtp53 or R175H mutp53 protein was pulled down by anti-p53 (DO-1) beads, and the elutes were subjected to LC-MS/MS analysis. The potential wtp53 or R175H mutp53-interacting proteins are listed with the number of peptides identified by LC-MS/MS analysis.

Supplemental Table 2

Tumor spectrum of *TRIM21*$^{+/+}$ *p53*$^{R172H/R172H}$ and *TRIM21*$^{-/-}$ *p53*$^{R172H/R172H}$ mice

Tumor type	*TRIM21*$^{+/+}$ *p53*$^{R172H/R172H}$ (n=22 mice)	*TRIM21*$^{-/-}$ *p53*$^{R172H/R172H}$ (n=24 mice)
Lymphoma	18	17
Osteosarcoma	1	1
Liposarcoma	0	1
Spindle cell sarcoma/ sarcoma	1	1
Angioma/ angiosarcoma	3	5
Carcinoma	1	1
Number of metastasis	0	2
Total number of tumors	24	28

Supplemental Figures

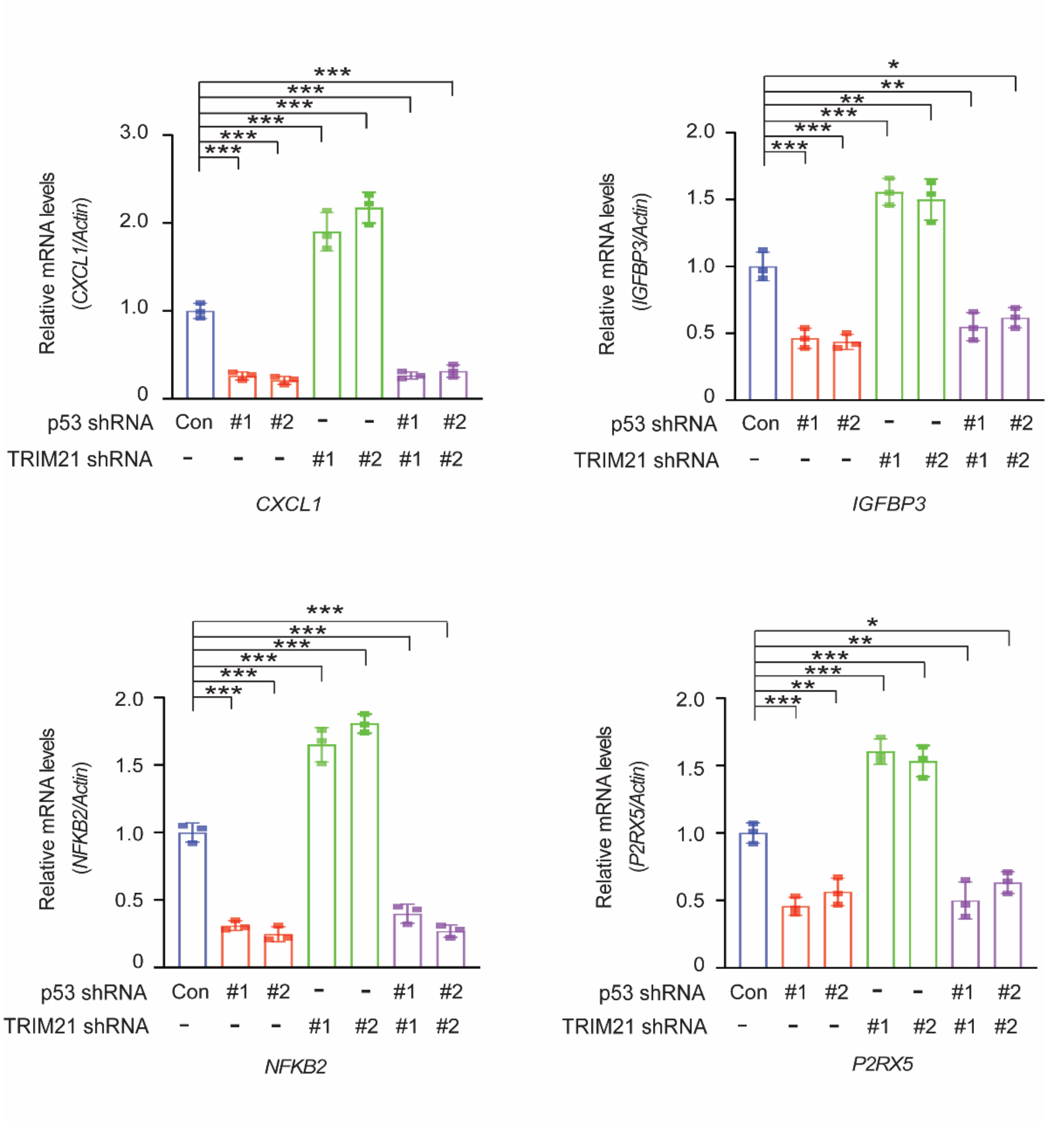

Supplemental Figure 1. TRIM21 negatively regulates the expression of mutp53-regulated genes in a largely mutp53-dependent manner. TRIM21 and/or R175H mutp53 were knocked down by 2 different shRNA vectors in SK-BR3 cells. The expression of mutp53-regulated genes, including *CXCL1, IGFBP3, NFKB2* and *P2RX5*, was measured by Taqman real-time PCR assays and normalized with *Actin*. Data are presented as mean ± S.D. ($n = 3$). Statistical analysis was performed using one-way ANOVA followed by Dunnett's test. *: p<0.01; **: p<0.001; ***: p<0.0001.

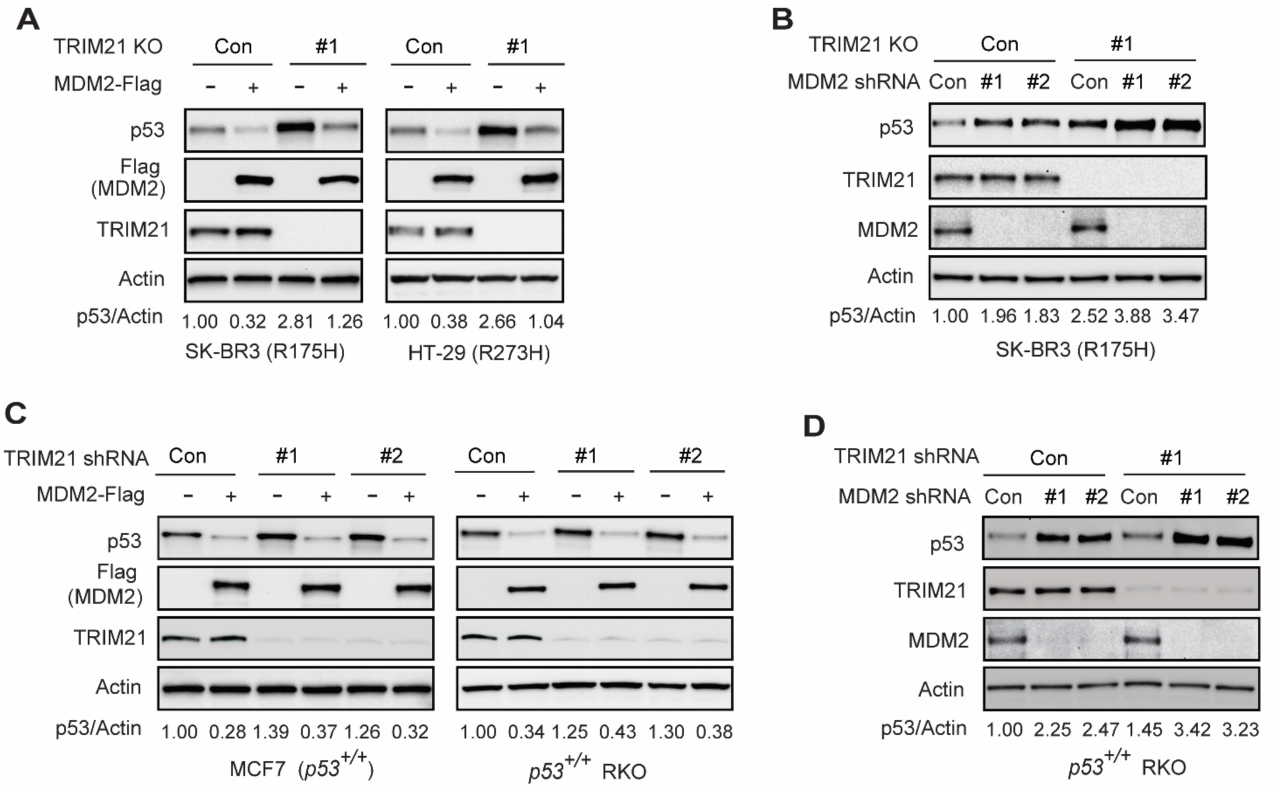

Supplemental Figure 2. MDM2 negatively regulates mutp53 and wtp53 protein levels in a TRIM21-independent manner in cancer cells. (**A**) Ectopic MDM2-Flag expression decreased endogenous mutp53 protein levels in both SK-BR3 and HT29 cells with or without TRIM21 KO. (**B**) MDM2 knockdown increased endogenous mutp53 protein levels in SK-BR3 cells with or without TRIM21 KO. MDM2 was knocked down by 2 different shRNA vectors. (**C**) Ectopic MDM2-Flag expression decreased endogenous wtp53 protein levels in both MCF7 and *p53*$^{+/+}$ RKO cells with or without TRIM21 knockdown. TRIM21 was knocked down by 2 different shRNA vectors. (**D**) MDM2 knockdown increased endogenous wtp53 protein levels in *p53*$^{+/+}$ RKO cells with or without TRIM21 knockdown. p53 protein levels in cells were analyzed by Western-blot assays.

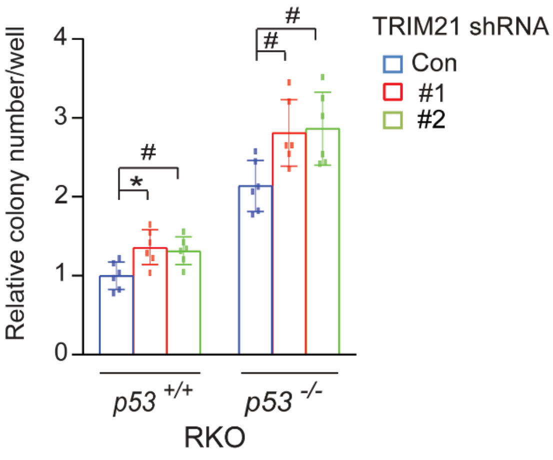

Supplemental Figure 3. The effect of TRIM21 knockdown on the anchorage-independent growth of *p53*$^{+/+}$ and *p53*$^{-/-}$ RKO cells. *p53*$^{+/+}$ RKO and its isogenic *p53*$^{-/-}$ RKO cells with or without TRIM21 knockdown by 2 different shRNA vectors were used for the anchorage-independent growth in soft agar. Data are presented as mean ± S.D. ($n = 6$). Statistical analysis was performed using one-way ANOVA followed by Dunnett's test. #: p<0.05; *: p<0.01.

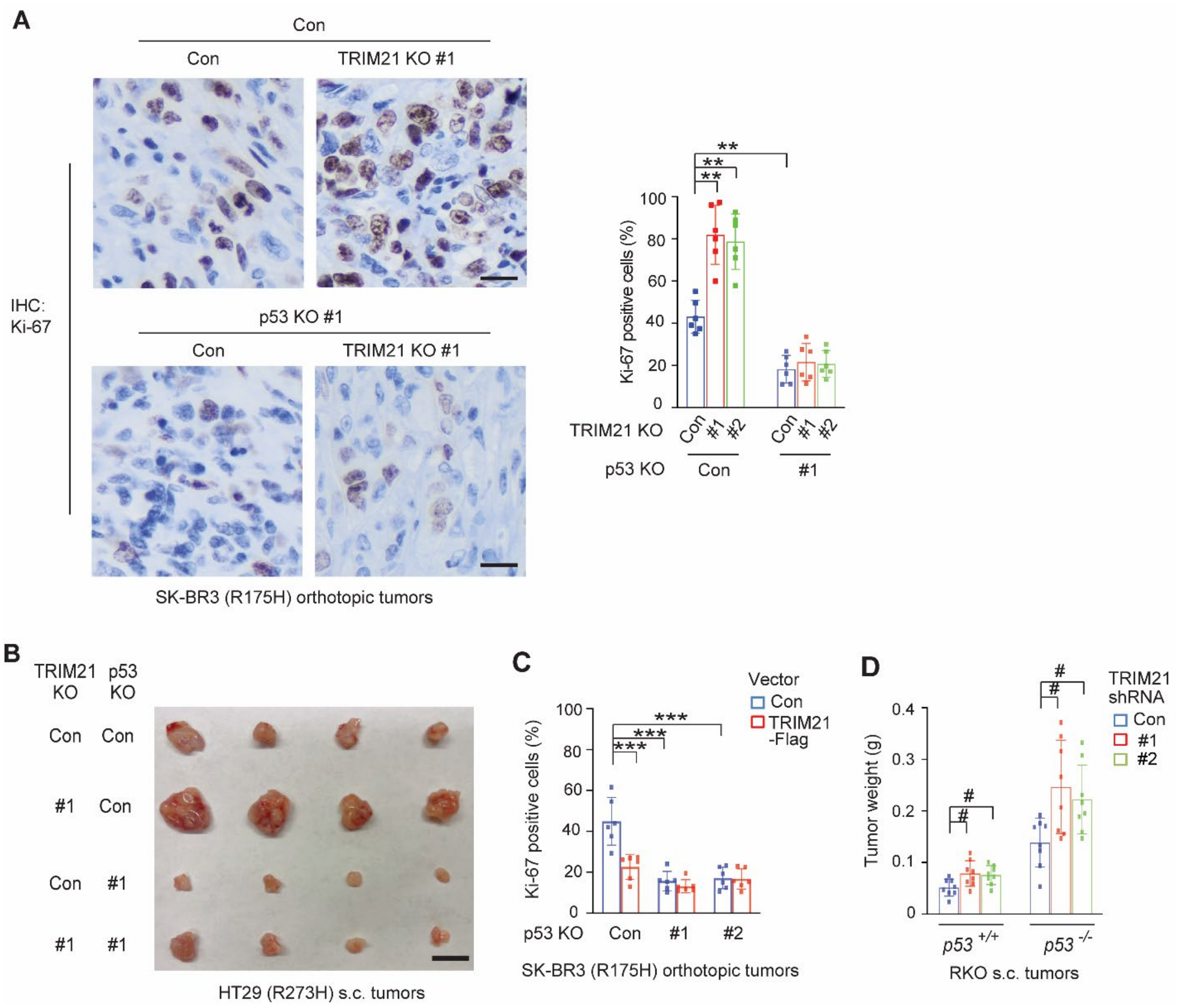

Supplemental Figure 4. The effect of TRIM21 on the growth of xenograft tumors in mice. (**A**) IHC staining of Ki-67 in SK-BR3 orthotopic tumors as described in Figure 5A. Left panel: representative images of IHC staining. Scale bar: 20 µm. Right panel: The percentage of Ki-67-positive cells in SK-BR3 tumors. Data are presented as mean ± S.D. ($n = 6$). **: $p<0.001$; Two-way ANOVA followed by Dunnett's test or Bonferroni's test. (**B**) TRIM21 KO in HT29 cells promoted the growth of s.c. xenograft tumors formed by cells, which was greatly abolished by R273H mutp53 KO in cells. Presented are representative images of the collected tumors as described in Figure 5D. Scale bar: 10 mm. (**C**) Percentage of Ki-67-positive cells in orthotopic breast tumors formed by control or mutp53 KO SK-BR3 cells with or without TRIM21-Flag expression as described in Figure 5F. Ki-67-positive cells were examined by IHC staining. Data are presented as mean ± S.D. ($n = 6$). ***: $p<0.0001$; Two-way ANOVA followed by Dunnett's test or Bonferroni's test. (**D**) $p53^{+/+}$ RKO and $p53^{-/-}$ RKO cells with or without TRIM21 knockdown by 2 different shRNA vectors were used for s.c. xenograft tumorigenesis assays in mice. Data are presented as mean ± S.D. n = 8 mice/group. Statistical analysis was performed using one-way ANOVA followed by Dunnett's test. #: $p<0.05$.

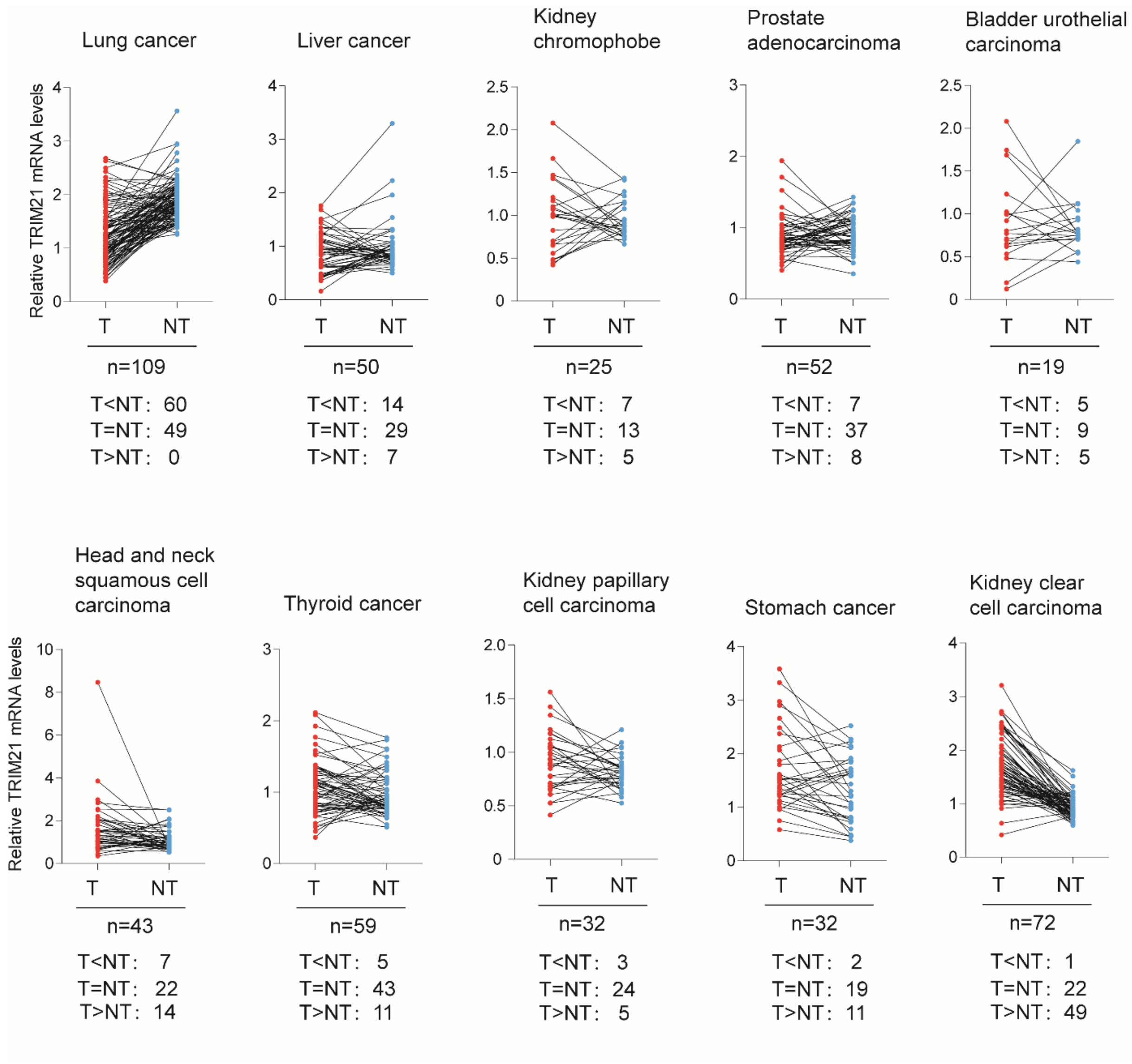

Supplemental Figure 5. The mRNA expression of *TRIM21* in different types of human cancers. Comparison of *TRIM21* mRNA levels between tumors (T) and matched adjacent non-tumor tissues (NT) in different types of cancers. The pairs were divided into three groups (T<NT, T=NT, and T>NT) using ±1.5-fold expression difference of *TRIM21* between tumors and non-tumor tissues as a cut-off level. The number of each group was listed. The data were obtained from TCGA.

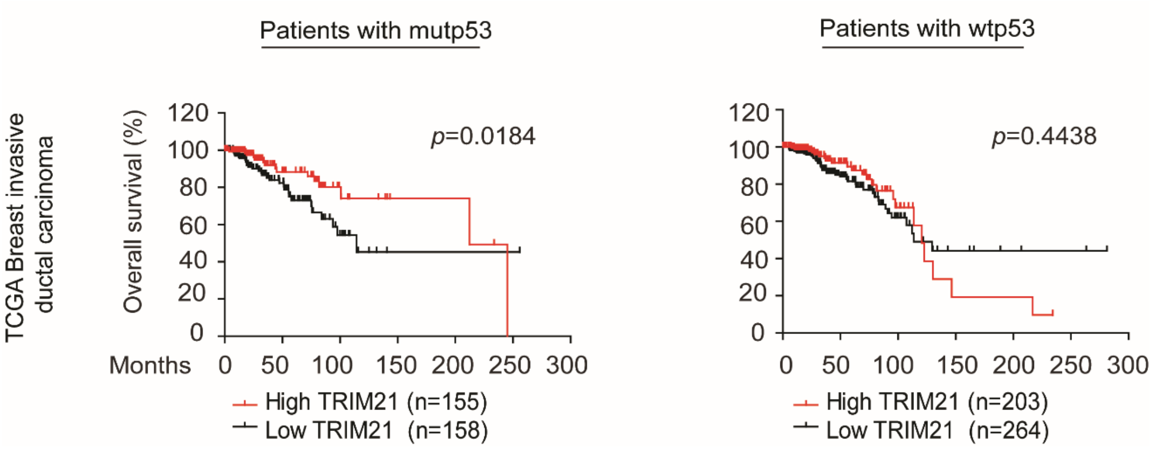

Supplemental Figure 6. Low *TRIM21* mRNA expression is associated with poor overall survival in patients with breast invasive ductal carcinomas carrying mutp53. Kaplan-Meier survival analysis was employed to analyze the relationship between *TRIM21* mRNA expression and the overall survival of patients with breast invasive ductal carcinomas carrying mutp53 or wtp53. The survival information and *TRIM21* expression z-score relative to normal samples were obtained from cBioPortal. The patients were divided into low or high *TRIM21* expression groups according to the cut-off of z-score = 0. Kaplan-Meier curves were drawn by using GraphPad Prism software, and differences between the two survival curves were analyzed using the log-rank (Mantel-Cox) test.

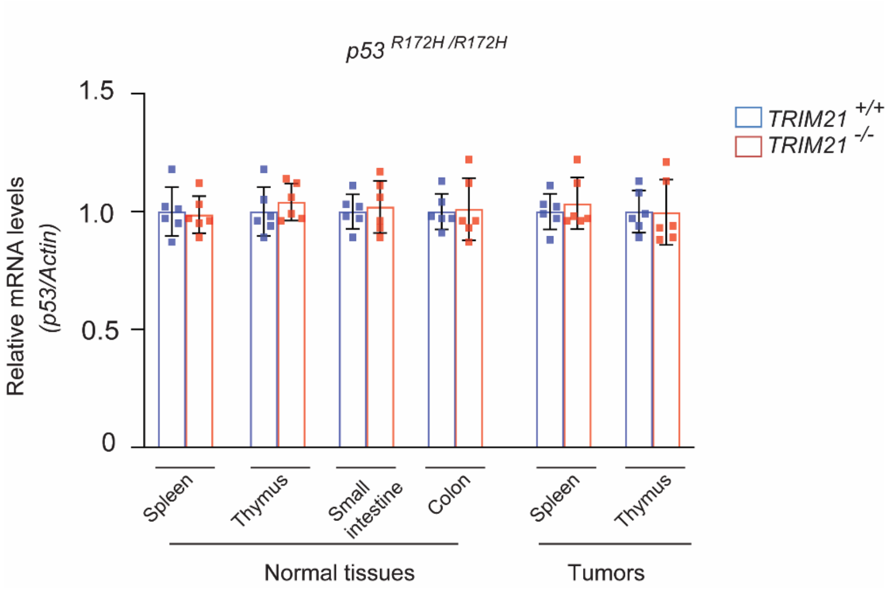

Supplemental Figure 7. TRIM21 does not affect the mRNA levels of *mutp53* in normal tissues or tumors in *p53*$^{R172H/R172H}$ mice. Normal tissues, including the spleen, thymus, small intestine and colon, as well as tumors, including the splenic and thymic lymphomas, in *TRIM21*$^{+/+}$ *p53*$^{R172H/R172H}$ and *TRIM21*$^{-/-}$ *p53*$^{R172H/R172H}$ mice were used for analysis. The mRNA levels of *mutp53* were measured by Taqman real-time PCR assays and normalized with *Actin*. No significant difference in the *mutp53* mRNA levels was observed between *TRIM21*$^{+/+}$ *p53*$^{R172H/R172H}$ and *TRIM21*$^{-/-}$ *p53*$^{R172H/R172H}$ mice. Data are presented as mean ± S.D. (n = 6 tumors/group). Statistical analysis was performed using unpaired Student's *t*-test.
